# Genotype/Phenotype Relationship: Lessons From 137 Patients With PMM2-CDG

**DOI:** 10.1155/2024/8813121

**Published:** 2024-10-03

**Authors:** Sander Pajusalu, Mari-Anne Vals, Mercedes Serrano, Peter Witters, Anna Cechova, Tomáš Honzik, Andrew C. Edmondson, Can Ficicioglu, Rita Barone, Pascale De Lonlay, Claire-Marine Bérat, Sandrine Vuillaumier-Barrot, Christina Lam, Marc C. Patterson, Mirian C. H. Janssen, Esmeralda Martins, Dulce Quelhas, Jolanta Sykut-Cegielska, Jehan Mousa, Roser Urreizti, Peter McWilliams, Frederique Vernhes, Horacio Plotkin, Eva Morava, Katrin Õunap

**Affiliations:** ^1^Genetics and Personalized Medicine Clinic, Tartu University Hospital, L. Puusepa Street 2, Tartu, Estonia; ^2^Department of Genetics and Personalized Medicine, Institute of Clinical Medicine, University of Tartu, L. Puusepa Street 2, Tartu, Estonia; ^3^Children's Clinic, Tartu University Hospital, N. Lunini Street 6, Tartu, Estonia; ^4^Pediatric Neurology Department and Clinical Biochemistry and Genetics Units, Hospital Sant Joan de Déu, Institut de Recerca Sant Joan de Déu, Barcelona, Spain; ^5^U-703 Centre for Biomedical Research on Rare Diseases (CIBER-ER), Instituto de Salud Carlos III, Barcelona, Spain; ^6^Department of Paediatrics, Metabolic Disease Center, University Hospitals Leuven, Leuven, Belgium; ^7^Department of Development and Regeneration, Faculty of Medicine, KU Leuven, Leuven, Belgium; ^8^Department of Pediatrics and Inherited Metabolic Disorders, First Faculty of Medicine, Charles University and General University Hospital in Prague, Prague, Czech Republic; ^9^Division of Human Genetics, Department of Pediatrics, Children's Hospital of Philadelphia, Pennsylvania, USA; ^10^Child Neuropsychiatry Unit, Department of Clinical and Experimental Medicine, University of Catania, Catania, Italy; ^11^Research Unit of Rare Diseases and Neurodevelopmental Disorders, Oasi Research Institute-IRCCS, Troina, Italy; ^12^Reference Center for Inborn Errors of Metabolism, Necker Hospital, APHP, University of Paris, Inserm UMR_S1163, INEM, and Institut Imagine, Filière G2M, MetabERN, Paris, France; ^13^Biochemistry and Genetics Department, Bichat-Claude Bernard Hospital, AP-HP, University of Paris and Inserm U1149, Paris, France; ^14^Center for Integrative Brain Research, Seattle Children's Research Institute, Seattle, Washington, USA; ^15^Division of Genetic Medicine, Department of Pediatrics, University of Washington School of Medicine, Seattle, Washington, USA; ^16^Departments of Neurology and Pediatric and Adolescent Medicine, Mayo Clinic, Rochester, Minnesota, USA; ^17^Department of Internal Medicine, Radboud University Medical Centre, Nijmegen, Netherlands; ^18^Centro de Genética Médica, Centro Hospitalar Universitário de Santo António, Porto, Portugal; ^19^Department of Inborn Errors of Metabolism and Paediatrics, The Institute of Mother and Child, Warsaw, Poland; ^20^Department of Clinical Genomics, Mayo Clinic, Rochester, Minnesota, USA; ^21^Glycomine, Inc., San Francisco, California, USA; ^22^Department of Laboratory Medicine and Pathology, Mayo Clinic, Rochester, Minnesota, USA; ^23^Department of Genetics and Genomics Sciences, Icahn School of Medicine at Mount Sinai, New York City, New York, USA

**Keywords:** congenital disorders of glycosylation, genetic variations, genotype/phenotype correlations, inherited metabolic disorders, natural history, phosphomannomutase 2-CDG, PMM2

## Abstract

We report on the largest single dataset of patients with PMM2-CDG enrolled in an ongoing international, multicenter natural history study collecting genetic, clinical, and biological information to evaluate similarities with previous studies, report on novel findings, and, additionally, examine potential genotype/phenotype correlations. A total of 137 participants had complete genotype information, representing 60 unique variants, of which the most common were found to be p.Arg141His in 58.4% (*n* = 80) of participants, followed by p.Pro113Leu (21.2%, *n* = 29), and p.Phe119Leu (12.4%, *n* = 17), consistent with previous studies. Interestingly, six new variants were reported, comprised of five missense variants (p.Pro20Leu, p.Tyr64Ser, p.Phe68Cys, p.Tyr76His, and p.Arg238His) and one frameshift (c.696del p.Ala233Argfs∗100). Patient phenotypes were characterized via the Nijmegen Progression CDG Rating Scale (NPCRS), together with biochemical parameters, the most consistently dysregulated of which were coagulation factors, specifically antithrombin (below normal in 79.5%, 93 of 117), in addition to Factor XI and protein C activity. Patient genotypes were classified based upon the predicted pathogenetic mechanism of disease-associated mutations, of which most were found in the catalysis/activation, folding, or dimerization regions of the PMM2 enzyme. Two different approaches were used to uncover genotype/phenotype relationships. The first characterized genotype only by the predicted pathogenic mechanisms and uncovered associated changes in biochemical parameters, not apparent using only NPCRS, involving catalysis/activation, dimerization, folding, and no protein variants. The second approach characterized genotype by the predicted pathogenic mechanism and/or individual variants when paired with a subset of severe nonfunctioning variants and uncovered correlations with both NPCRS and biochemical parameters, demonstrating that p.Cys241Ser was associated with milder disease, while p.Val231Met, dimerization, and folding variants with more severe disease. Although determining comprehensive genotype/phenotype relationships has previously proven challenging for PMM2-CDG, the larger sample size, plus inclusion of biochemical parameters in the current study, has provided new insights into the interplay of genetics with disease.

**Trial Registration:** NCT03173300.

## 1. Introduction

Congenital disorders of glycosylation (CDG) are inborn errors of metabolism caused by alterations to enzymatic processes of carbohydrate (glycan) formation, assembly, and attachment to proteins and/or lipids. PMM2-CDG is one of more than 160 known CDGs [1–3]. PMM2-CDG is the most common N-linked glycosylation defect representing more than 60% of all cases [4].

PMM2-CDG is inherited in an autosomal recessive pattern, with most cases involving biallelic compound heterozygous variants in *PMM2*. The clinical presentation and course of the disorder are highly variable, ranging from infants who die in the first weeks of life to patients surviving into adulthood. Clinical and metabolic presentations tend to be similar in siblings [5–7]. PMM2-CDG is suspected based on a Type 1 transferrin isoform pattern measured in blood and confirmed by identification of biallelic pathogenic variants in *PMM2* by molecular genetic testing. Enzymatic activity can be used as part of the diagnostic confirmation; however, there are patients carrying biallelic pathogenic *PMM2* variants presenting with classic clinical features and high to normal residual enzyme activity in blood [8].

PMM2-CDG is caused by deficient PMM2 enzyme activity, the enzyme responsible for conversion of mannose-6-phosphate (M6P) to mannose-1-phosphate (M1P), resulting in hypoglycosylation of N-linked glycoproteins [9–11]. The PMM2 enzyme is only active as a dimer, and each chain is comprised of two domains connected through a flexible polypeptide sequence (hinge peptides), a cap region, and a catalytic domain [12]. The activator binding domain binds the distal phosphate group on the C-6 position of M6P as a phosphate group is added to the C-1 position. The cap region folds to envelop the substrate as the enzymatic reaction proceeds. After ligand binding, a conformational change creates an environment for catalysis [13, 14].

*PMM2*, which encodes the PMM2 enzyme, is located in chromosomal region 16p13.2 [11, 15]. Numerous pathogenic variants have been identified in *PMM2* [16]. The p.Arg141His variant is the most common single pathogenic variant reported in PMM2-CDG [17] and, indeed, has a carrier frequency of 1/126 in the general population and 1/92 in Europeans, as reported in the gnomAD database [18]. This particular genetic variation affects the activator binding domain of the enzyme, leading to a complete loss of function, and is always found in combination with another destabilizing variant in affected individuals. Despite its prevalence, this variant has never been reported in the homozygous state, suggesting that this genotype is incompatible with life [19]. Less deleterious variants have been reported in homozygous form in patients, including a patient with uniparental disomy [20]. Notwithstanding multiple efforts, a comprehensive genotype/phenotype correlation has not been found, probably due to the large number of variants and combinations in heterozygosity and the low population frequency of some of the variants.

The aim of this study was to collect a large international dataset of genotype and phenotype data of PMM2-CDG patients to evaluate similarities with previous studies, report on novel findings, and, additionally, examine potential genotype/phenotype correlations.

## 2. Material and Methods

### 2.1. Study Design and Participants

Genetic testing, clinical, and biological information were prospectively collected in PMM2-CDG patients as part of an ongoing international, multicenter natural history study. This study was conducted at 11 sites, eight in Europe (Belgium, Czech Republic, France, Italy, the Netherlands, Portugal, Poland, and Spain) and three in the United States. Individuals included in this report were diagnosed with PMM2-CDG as documented by genetic study of biallelic pathogenic variants in *PMM2* and/or PMM2 enzymatic activity and were willing and able to adhere to the study assessments and schedule. Individuals with other CDGs were excluded from this study.

Enrolled participants were assessed for signs and symptoms of PMM2-CDG by conducting a thorough medical history review, physical examination, laboratory testing, and imaging studies at the beginning of the study and approximately every 6 months for up to 4 years. This study did not offer any new therapy or changes to the participants' routine. No randomization was performed.

### 2.2. Outcome Measures

The primary objective of the current natural history study was to collect information in patients with PMM2-CDG to determine an appropriate set of clinical and biological parameters to be used in subsequent interventional clinical trials. The data collected includes assessments of developmental, neurologic, ophthalmologic, audiologic, immunologic, hematologic, dermatologic, cardiac, pulmonary, gastrointestinal, hepatic, renal, endocrine, and musculoskeletal manifestations.

### 2.3. Study Assessments

Study assessments were based on the standard of care at each respective site. All analyses shown in this paper were performed on data collected at the initial (baseline) study visit; however, the authors made efforts to collect additional historical information for the participants with novel variants not previously published.

The clinical disease severity was evaluated using the Nijmegen Progression CDG Rating Scale [21, 22], which evaluates PMM2-CDG in three domains: Section 1, current function; Section 2, system-specific involvement; and Section 3, current clinical assessment, with higher scores denoting more evident clinical abnormalities. For each evaluation within a section, there are four possible responses reflecting *normal* (0), *mild* (1), *moderate* (2), and *severe* (3) impairment, with one exception in Section 3 in the form of a flow chart to assess developmental progress for which a score of 0–7 is possible [21]. In this study, the ranges used for the domains were current function (0–15 without the self-care and education items to harmonize all age groups), system-specific involvement (score ranges 0–30), and current clinical assessment (score ranges 0–31) for total scores ranging from 0 to 76.

One of the goals of this study was to augment the NPCRS clinical evaluations with relevant biochemical measurements and, thereby, provide a more complete description of the phenotype. To this end, we focused on a subset of the most commonly assessed and disease-specific laboratory measures, specifically coagulation (antithrombin activity, protein C activity [PCA], Factor IX activity, and Factor XI activity), liver function (alkaline phosphatase [ALP], alanine transaminase [ALT], aspartate aminotransferase [AST], and ceruloplasmin [CERP]), metabolic (insulin-like growth factor 1 [IGF-1], insulin-like growth factor binding protein 3 [IGFBP3]), and serum glycosylated transferrin ratios (a-oligo/di-oligo and mono-oligo/di-oligo).

Genetic variants in *PMM2* were classified into seven activity categories based on the predicted pathogenic mechanism [23]. These categories reflect regions of importance for the function and/or the stability of the enzyme. In particular, certain variants are expected to be deactivating if they interfere with substrate (M1P) binding or the catalytic role of Mg^2+^. Additionally, since the enzyme is only active as a dimer, variants in the binding interface between the two individual protein subunits, as well as those that reduce the ability of the protein to fold and envelop the substrate as the enzymatic reaction proceeds, can be deactivating. In all, Briso-Montiano et al. [23] define seven activity categories: catalysis or activator binding (C), dimerization (D), folding (F), folding without activity (FWO), Linker-2 (L), no protein (NP), and uncertain (U). Each participant was assigned to a respective activity category pairing based upon these seven individual categories and their respective predicted functional effect, creating 22 distinct activity category pairings. For instance, a participant with pathogenic variants p.Arg141His/p.Pro113Leu was assigned the activity category pairing catalysis/activator binding-dimerization (C-D).

### 2.4. Statistical Analyses

Demographic, background information, and clinical characteristics were summarized with descriptive statistics (means, standard deviations, medians, ranges for continuous variables, counts, and percentages for categorical variables).

Two analyses were conducted to evaluate potential genotype/phenotype correlations. The first analysis looked at the relationship between the activity category pairs that define each participant's genotype and their associated clinical characteristics and laboratory results (phenotype). Only activity category pairs with at least four participants, excluding any pair with the U category, were used for this analysis, resulting in eight (out of the potential 22) unique activity category pairs, namely, C-C, C-D, C-F, C-FWO, C-NP, D-FWO, D-NP, and F-F.

The second analysis looked at the relationship between various individual variants and/or activity categories when paired with a subset of severe nonfunctioning variants (p.Arg141His, p.Asp188Gly, p.Phe157Ser, p.Thr237Met, or p.Thr237Arg) and the patient's associated clinical characteristics and laboratory results. In particular, two individual variants, p.Cys241Ser and p.Val231Met, and the two activity categories, D (with *n* = 34 comprised of the three variants p.Pro113Leu, p.Phe119Leu, and p.Als108Val) and F (with *n* = 29 comprised of 10 variants, of which the three most prevalent variants in the category, namely, p.Val231Met, p.Val129Met, and p.Ile132Thr represented 58.6% of the total), were analyzed when paired with the severe nonfunctioning variant subset. This analysis is an expansion of the analysis conducted by Vaes et al. [12].

Continuous variables, such as NPCRS overall score, or laboratory results were analyzed using the nonparametric Kruskal–Wallis test to compare activity category pairs in the first analysis and groupings in the second analysis. Sex and age were checked for category or grouping dependence, and if necessary, a generalized linear model was used to correct for either sex or age in the comparisons. If the Kruskal–Wallis test was significant in the first analysis, a contrast analysis was performed to compare activity category pairs with results that stood out from the rest of the activity category pairs. When performing multiple comparisons, *p* values were adjusted using the Benjamini and Hochberg false discovery rate method [24]. All tests were two-sided at the 5% significance level. All analyses were conducted using SAS(R) Version 9.4 (http://www.sas.com), R Version 4.3.1 (2023-06-16), and Rstudio Version 2023.06.1.

### 2.5. Study Oversight

The study was conducted in accordance with the Declaration of Helsinki and Good Clinical Practice. Institutional Review Boards at each study site approved the protocols and informed consent forms. All parents, caregivers, or participants provided written informed consent before enrollment.

## 3. Results

### 3.1. Study Population

A total of 139 individuals enrolled in this study. Of these, 137 participants had complete genotype information and ranged in age from 0.5 to 68.4 years, with the median age of 10.4 years (Table 1). Males comprised 53% and females 47%. The demographic characteristics (age and sex) of the participants included in each of the two genotype/phenotype analyses were similar.

The distribution of the demographic characteristics by country and region is shown in Tables S1 and S2, respectively, showing that the US sites tended to have a younger population as compared to the EU with a median age of 6.6 versus 12.80 which was statistically significant (*p* = 0.0079).

**Figure 1:**
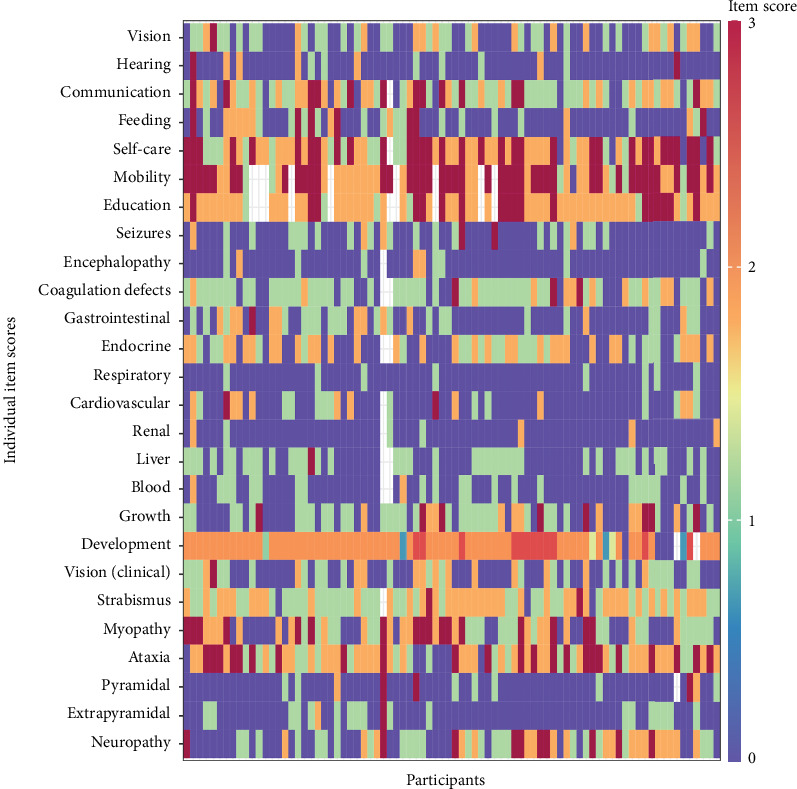
Heat map showing the scores for all participants as indicated by each rectangle, which represents one participant for each NPCRS item (a higher score reflects more disease severity). Thus, when the scores are viewed together, the severity of impairment can be seen. For example, mobility tends to be mostly red and orange, indicating severe mobility impairment in most patients.

The clinical presentation of participants was heterogeneous (Table 2). Looking at the clinical signs within the individual NPCRS item scores with a heat map (Figure 1), the areas most affected were communication, self-care, mobility, education, development, strabismus, myopathy, ataxia, and neuropathy; coagulation defects, gastrointestinal and endocrine abnormalities, were also common.

The most common biochemical findings at baseline (Table 3) were coagulation lab abnormalities, specifically low antithrombin (79.5%, 93 out of 117), followed by PCA (56.8%, 54 out of 95) and Factor XI (56.1%, 64 out of 114). Interestingly, Factor IX levels were mostly within the normal range (84.6%, 88 out of 104).

Serum transferrin ratios (which were collected by a subset of seven sites, totaling 59 participants) were also outside of the normal range as may be expected since it is often the first diagnostic test used if a diagnosis of PMM2-CDG is suspected. The a-oligo/di-oligo is above the upper limit of normal in 78% of the participants (46 out of 59, 0.022), while the mono-oligo/di-oligo is above the upper limit of normal in 88% of the participants (52 out of 59, 0.1).

IGF-1 and IFBP3 tended to be on the lower side, with almost a quarter of the results below the lower limit of normal and only a few results above the upper limit of normal (one and two participants, respectively, for IGF-1 and IGFBP3).

Liver function tests indicate some elevated ALP for age in 17.2% (20 out of 116) participants. ALT was above the upper limit of normal for 45 out of 130 participants (34.6%). For AST, over one-third (36.3%, 45 out of 124) were above the upper limit of normal. For CERP, 16 out of 82 individuals (19.5%) were below the lower limit of normal (with limits of normal used from local laboratories).

For the genotype/phenotype analyses, each variant was assigned to an activity category based upon their respective pathogenic mechanisms (see Material and Methods). The activity category U was excluded from all analyses, and, for the first genotype/phenotype analysis (classifying genotype using activity pairs only), those activity category pairs with less than four participants were excluded. These exclusion conditions resulted in a total of 88 participants (Table S3) for the first analysis. For the second genotype/phenotype analysis (classifying genotype with one variant from a subset of severe nonfunctioning variants, combined with specific individual variants or activity categories), there were 102 participants, and, following the analysis of Vaes et al. [12], six were excluded because they had two of the nonfunctioning variants, and this resulted in 96 participants (Table S4).

### 3.2. Genetic Variants

A total of 60 unique variants were found (Table S5). The majority (58.4%) of participants (*n* = 80) had the p.Arg141His variant (Table 4). The next most frequently seen were p.Pro113Leu (*n* = 29, 21.2%) and p.Phe119Leu (*n* = 17, 12.4%). Most of the pathogenic variants were located in the C, F, or D regions of the gene (Table 5). Variants in the C region were found in 98 participants (71.5%), while variants affecting D were found in 49 participants (35.8%). Variants affecting F were found in 47 participants (34.3%), and FWO was found in 26 patients (19.0%). Variants leading to complete loss of function (NP) were found in 17 participants (12.4%), and variants affecting the L region were less common (*n* = 6, 4.4%). Thirty-two participants (23.4%) had pathogenic variants with an U functional consequence.

Six participants were homozygous: p.Phe119Leu (*n* = 2), p.Gly15Ala (*n* = 1), p.Lys51Arg (*n* = 1), p.Pro113Leu (*n* = 1), and p.Gly214Ser (*n* = 1). Two participants with homozygous p.Pro113Leu and p.Phe119Leu, respectively, had maternal uniparental disomy of Chromosome 16. These individuals were previously reported elsewhere [20]. One participant carried three pathogenic variants: p.Thr118Ser, p.Arg162Trp, and p.Pro184Thr. However, p.Thr118Ser and p.Pro184Thr variants were previously reported on the same allele [25]. p.Pro184Thr is in the L category, and both p.Arg162Trp and p.Thr118Ser are in the U category, so this trio of variants was classified in the L-U variant pair category.

No significant differences in variant frequencies were found between the US and EU patient cohorts (Tables S6 and S7). The p.Arg141His variant was the most prevalent variant in both. Interestingly, p.Arg162Trp was found only in the EU cohorts, being reported from different sites across Europe. p.Val231Met was more frequent in Poland compared to Western European sites and the United States.

**Figure 2:**
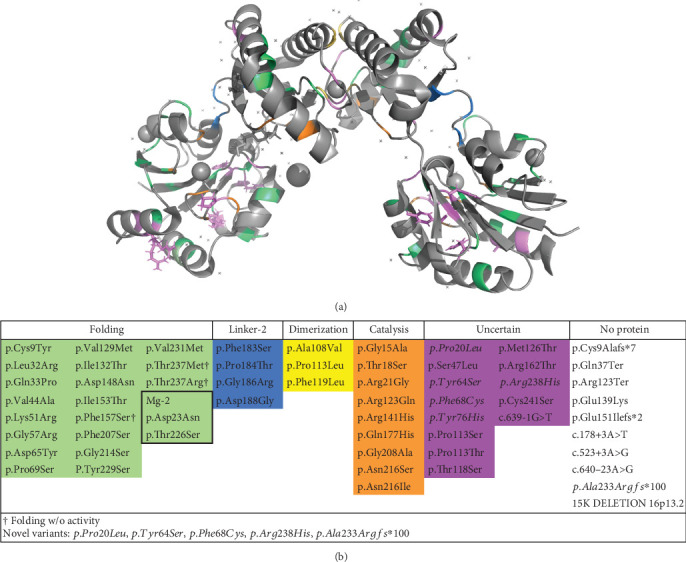
Structure of *PMM2* by variant analysis using the model from Briso-Montiano et al. [23]. PyMol2 software has been used to highlight the mutated residues. (a) Front view of the dimer with the 60 pathogenic variants in colors according to their categorization (see (b)). (b) Categorization of the 60 pathogenic variants identified in the natural history study.

Localization of previously described and new variants (discussed in the next section) at the protein level and protein alignment of nonreported variants in model organisms are represented in Figure 2.

### 3.3. Novel Genetic Variants

Six variants, not previously described in PMM2-CDG, were found, comprised of five missense variants (p.Pro20Leu, p.Tyr64Ser, p.Phe68Cys, p.Tyr76His, and p.Arg238His) and one frameshift (c.696del p.Ala233Argfs∗100). NPCRS and biochemical information on the patients containing these novel variants shows they presented with a range of disease severity (Table S8). The p.Arg238His (with the p.Arg141His) variant presented with a relatively mild phenotype, having coagulation and transferrin ratios in a normal range and an NPCRS of 18, as did the p.Tyr64Ser (with the p.Tyr21Gly) variant, having antithrombin and PCA dysregulated, Factor XI in a normal range, and an NPCRS of 10. However, the p.Pro20Leu (with p.Pro113Leu variant) presented with a more severe phenotype, having antithrombin, protein C, and Factor XI dysregulated, and an NPCRS of 29.

Additional biochemical information in the patients' medical history was sought to further understand these novel variants. Enzymatic activity analyses were reported on four of the six novel variants. The two variants p.Pro20Leu and p.Tyr76His (with the p.Pro113Leu and p.Thr237Met variants, respectively) showed partial to deficient enzymatic activity using the PMM2 activity assay. The variant p.Phe68Cys (with the p.Arg141His variant) had PMM2 activity of 211 nmol/h/mg in fibroblasts (reference range ≥ 700). Spectrophotometric examination of PMM2 and PMI (phosphomannose isomerase) activities in isolated lymphocytes showed 0.002 nmol/min/mg protein (controls 0.81–1.65) for PMM2, 17.5 nmol/min/mg protein for PMI (controls 7.83–21.0), and 0.00014 (controls 0.02–0.18) for the ratio PMI/PMM2. No enzymatic activity analysis was available for p.Tyr64Ser or p.Arg238His. All novel variants were classified as likely pathogenic based on ACMG criteria [26]. Specifically, the frameshift variant was classified as likely pathogenic based on PVS1 (loss-of-function variant) and PM2 criteria (very rare or absent in the general population, consistent with autosomal recessive inheritance). The missense variants were classified as pathogenic based on the criteria PM1 (variant in a well-defined functional region or variant hot spot without benign variation), PM2 (very rare or absent in the general population, consistent with autosomal recessive inheritance), PP2 (a missense variant in a gene with a low rate of benign missense variation), and PP3 (multiple computational evidence supporting pathogenicity). In addition, p.Pro20Leu, p.Tyr64Ser, p.Tyr76His, and p.Arg238His all caused a novel missense change at an amino acid residue where a different missense change determined to be pathogenic has been seen before, and thus PM5 criterium was applied to further support pathogenicity.

### 3.4. First Genotype/Phenotype Analysis: Analysis of Variant Pair Activity Categories

**Figure 3:**
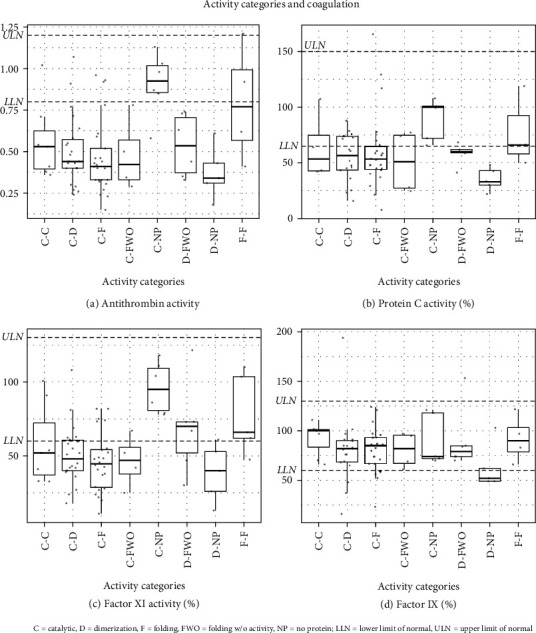
Coagulation parameters by activity categories for categories with at least four participants. (a) Antithrombin activity, (b) protein C activity, (c) Factor XI activity, and (d) Factor IX levels.

For the first genotype/phenotype analysis, genotype was described based only upon activity category pairs representing each participant (Table 6(a)), and although there were no statistically significant relationships between the resulting eight activity category pairs and NPCRS score (Table 6(b)), some activity category pairs were statistically different from all others with respect to a subset of the biochemical markers. Specifically, for antithrombin, the *p* value using the Kruskal–Wallis test was close to statistical significance, with a *p* value of 0.0541 (after FDR correction), and for Factor XI, the *p* value was 0.0384 (Table 6(c)), indicating that antithrombin and Factor XI were different for at least one of the activity category pairs. In fact, further examination of the results showed that for antithrombin and Factor XI, the C-NP and F-F categories had higher average values than the rest of the categories (Table 6(c)). A contrast analysis in a linear model with activity category as the independent variable was used to compare the C-NP and the F-F activity categories to the other categories combined. The difference in average antithrombin and Factor XI levels between the C-NP category and the other categories combined (excluding F-F) was calculated to be 0.42 (*p* < 0.0001) and 46% (*p* < 0.0001), respectively. For the F-F category, these differences (excluding C-NP) were 0.30 for antithrombin (*p* = 0.0081) and 28% for Factor XI (*p* = 0.0088) (Table 6(d)). In fact, for C-NP, the antithrombin and Factor XI average levels are well within the normal range, and for the F-F category, the antithrombin average levels are almost at the lower limit of normal while the average Factor XI levels are in the normal range (Figure 3). This analysis therefore suggests both these activity category pairs are associated with a milder coagulation phenotype.

**Figure 4:**
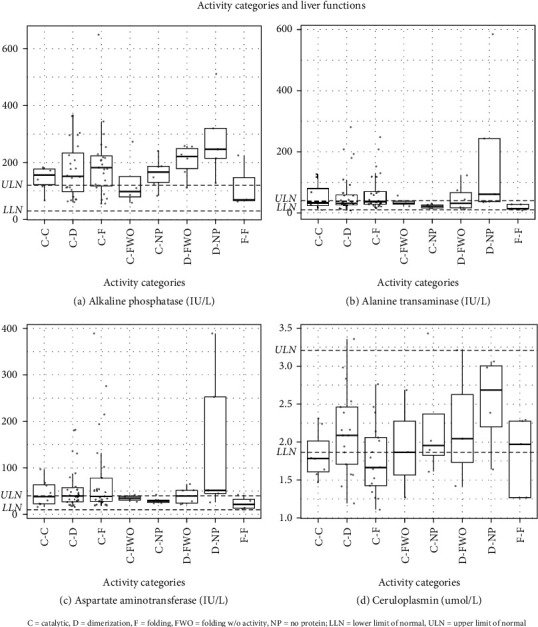
Liver function by activity categories for categories with at least four participants. (a) Alkaline phosphatase, (b) alanine transaminase, (c) aspartate aminotransferase, and (d) ceruloplasmin.

Among the liver parameters, ALT (the only other biochemical marker in which any activity category pair was statistically different from any others), the category that stood out was D-NP (Table 6(c)). Again, using a linear model with a contrast analysis, the difference in average levels between D-NP and the other category pairs combined was 68 IU/L (*p* value = 0.0002) (Table 6(e)). This difference suggests that this category pair results in a more severe liver phenotype (Figure 4). It is worth noting that there was no significant difference in age between the groups (Table 6(a)), so although liver enzymes are known to normalize with age, we can rule out this possibility as being a driver of the difference.

### 3.5. Second Genotype/Phenotype Analysis: Analysis of Participants With at Least One Nonfunctioning Variant

The second genotype/phenotype analysis was conducted to verify if the genotype/phenotype analysis reported by Vaes et al. [12] was observed in this larger population of individuals with PMM2-CDG and, further, to extend this analysis to new variants given the additional data available, as well as include key biochemical markers in addition to the NPCRS. To this end, the 96 participants with at least one of the nonfunctioning variants p.Arg141His, p.Phe157Ser, p.Thr237Arg, p.Thr237Met, and p.Asp188Gly were split into two groups for four subsequent analyses based upon the characteristics of the variant on the other allele (Table S9): (i) D category, (ii) F category, (iii) the variant p.Cys241Ser, and (iv) the variant Val.231Met (itself within the F category).

For the first analysis, (i) participants with one nonfunctioning variant combined with another variant in the D category were compared with participants (with one nonfunctioning variant) without a variant in the D category. The participants with a variant in the D category had higher NPCRS scores for Section 1, 3, and overall. Although the results were not statistically significant at the 5% level (5.9 vs. 4.5, *p* = 0.0798; 13.1 vs. 11.2, *p* = 0.0763; 22.9 vs. 20.1, *p* = 0.0984, respectively; Table 7), they nevertheless indicate a trend towards a more severe expression of PMM2-CDG.

For the second analysis, (ii) participants with one nonfunctioning variant combined with another variant in the F category were compared with participants (with one nonfunctioning variant) without a variant in the F category. The participants with a variant in the F category showed significantly lower values for antithrombin and Factor XI activity (0.44 vs. 0.60, *p* = 0.0316 and 44.6% vs. 62.8%, *p* = 0.0316, respectively) and significantly higher ALT (70.1 IU/L vs. 49.1 IU/L, *p* = 0.0364) (Table 8), and there was a trend in the a-oligo/di-oligo transferrin ratio, all suggesting that a F variant also resulted in a more severe expression of PMM2-CDG.

For the third analysis, (iii) participants with one nonfunctioning variant combined with the p.Cys241Ser were compared with participants (with one nonfunctioning variant) without a p.Cys241Ser variant. The first group, with the p.Cys241Ser variant, had significantly lower NPCRS Sections 1 (1.2 vs. 5.4, *p* = 0.0024), 3 (7.5 vs. 12.2, *p* = 0.0028), and overall scores (11.2 vs. 21.9, *p* = 0.0024; Table 9). With respect to laboratory assessments, the only significant result was for IGF-1, where on average, the group with p.Cys241Ser had significantly higher levels (254.93 ng/mL vs. 121.37 ng/mL, *p* = 0.0235; Table 9). However, there were also positive trends in the coagulation parameters antithrombin, Factor XI and Protein C, transferrin ratios, and AST. Taken together, the lower NPCRS scores and higher IGF-1, as well as trends in multiple other biochemical parameters for the group containing p.Cys241Ser, provide strong evidence of a milder expression of PMM2-CDG.

Finally, for the fourth analysis, (iv) participants with one nonfunctioning variant combined with the p.Val231Met were compared with participants (with one nonfunctioning variant) without a p.Val231Met variant. The p.Val231Met variant has been previously identified as a severe variant [27] and was the fourth most common in our overall dataset with 11 occurrences, equal to that of the p.Cys241Ser. Furthermore, this variant is contained within the activity category of F, which was found to imply a more severe phenotype when combined with a severe nonfunctioning variant in the current analysis. The conclusions that were found for the broader activity category of F (also including p.Val129Met, p.Ile132Thr, and seven other variants when in combination with a severe nonfunctioning variant) also held for the p.Val231Met by itself. Specifically, the coagulation factors PCA (mean = 36.75% vs. 65.1%, *p* value = 0.0260) and Factor XI activity (mean = 38.75% vs. 59.58%, *p* value = 0.0291) were significantly lower in Group 1 compared to Group 2, while the liver enzymes ALP (mean = 294.94 U/L vs. 168.96 U/L, *p* value = 0.0260), ALT (mean = 141.00 U/L vs. 46.80 U/L, *p* value < 0.0410), and aspartate transaminase (mean = 110.38 U/L vs. 48.5 U/L, *p* value = 0.0199) were still significantly higher in Group 1 compared to Group 2 (Table 10).

**Figure 5:**
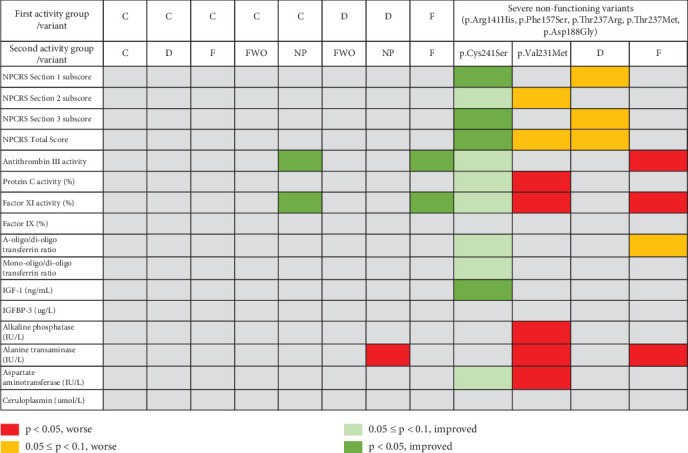
Heat map showing the genotype/phenotype relationships found in the current study.

In summary, when looking at activity category pairs, two of the coagulation parameters (antithrombin and Factor XI activity) had better levels for the F-F and catalysis-NP activity categories, and one of the liver parameters (ALT) showed worse levels in the D-FWO (Figure 5). These results show the utility of including biochemical parameters in the analysis, since none of these relationships are apparent by looking at the NPCRS alone. Additionally, using a different approach to determine genotype/phenotype relationships, namely, the combination of activity categories and/or individual variants with a subset of severe nonfunctioning variants, the two coagulation factors (antithrombin activity and Factor XI activity) and the liver function test ALT were affected negatively by the presence of a F variant. The p.Val231Met variant alone also negatively affected the coagulation parameters, Protein C and Factor XI activity, as well as three of the liver functions: ALP, ALT, and AST. Furthermore, the coagulation parameters, a liver parameter, and the transferrin ratios showed a positive trend, and IGF-1 levels were significantly higher values in combination with p.Cys241Ser. NPCRS levels observed with this variant were consistent with the biochemical findings, having significantly lower values, which is associated with milder disease, and, by contrast, there was a trend towards higher scores with D variants.

## 4. Discussion

The current study is a report of the largest single dataset of patients with PMM2-CDG. Our goals were to evaluate similarities with previous studies, report on novel findings, and, additionally, examine potential genotype/phenotype correlations. The PMM2 protein is a dimer, and most PMM2-CDG patients are compound heterozygous, carrying one allele encoding an inactive *PMM2* variant, such as p.Arg141His, and a second allele with a hypomorphic variant. The combination has variable effects on PMM2 protein stability and on heterodimer formation, with both contributing to the observed biochemical and phenotypic variation. We were looking for more comprehensive genotype/phenotype correlations than have been described in the past, based upon a greater volume of data and by adding a variety of biochemical markers into the analysis, combined with the NPCRS clinical severity scores that have been used previously. Additionally, we utilized eight different activity categories to classify genotypes for analysis of potential relationships with phenotype. We found that the addition of biochemical markers, particularly the coagulation parameters antithrombin and Factor XI, as well as transferrin ratios and liver enzymes, allowed us to uncover new relationships among activity categories (F-F, C-NP, and D-NP) as well as the individual variants p.Val231Met and p.Cys241Ser, and variants in the D and F category, when combined with a severe nonfunctioning variant (Table 5).

The most frequent single pathogenic variants in *PMM2* found in this study were p.Arg141His, p.Pro113Leu, and p.Phe119Leu, consistent with other studies [19]. Using an activity classification [23], the most frequent location for these variants was in the C, F, or D regions of the gene.

We observed an increased frequency of the p.Val231Met variant in Poland compared to sites in Western Europe and the United States. This agrees with previous results [28]. p.Val231Met has also been shown to be the second most common variant in Estonia, 23% among biobank participants [19, 29].

In our study, we identified six homozygotes, of which three were in the D, two in the F, and one in the C category. The p.Val231Met variant (classified in the F category) was observed in 11 patients but never in the homozygous state in our study; however, this variant has previously been reported to be homozygous in PMM2-CDG patients [30]. Interestingly, our study and others have identified this variant as resulting in a more severe phenotype, despite the fact it has been observed as a homozygote.

Limitations of this study include the fact that not all patients in each country were recruited, and not all countries in Europe are represented. Because only some individuals with PMM2-CDG participated from each country, no reliable conclusions on population frequencies could be obtained; therefore, recruited patients were not representative of the distribution of variants in each participating country. Moreover, the design of our study might have caused an underrepresentation of p.Arg141His and other severe variants since patients that died early in infancy were not among those evaluated.

### 4.1. Genotype/Phenotype Correlations

Recently, Vaes et al. [12] evaluated 17 different pathogenic variants in *PMM2* in 26 PMM2-CDG patients. They suggested that variants involving the D domain (p.Pro113Leu or p.Phe119Leu in their study) resulted in a significantly higher NPCRS Section 1, 3, and overall score (*p* = 0.0012, 0.0039, and 0.002, respectively) in the presence of one nonfunctioning allele and therefore a more severe clinical outcome, a finding that was supported in this study with the addition of p.Ala108Val in our D category. Although the results in our study did not reach statistical significance, they did show a similar trend in the same sections of the NPCRS. In the Vaes et al. [12] study, there were 11 participants in each of the comparison groups, whereas the current study included 34 in the D category and 62 in the comparison group, indicating that the initial finding becomes weaker upon closer examination with a larger sample size.

Vega et al. [31] further suggested a link between disease severity and genotype such that pathogenic variants in the *PMM2* gene that affect F or stability seem to be associated with a milder phenotype. They reported results obtained from Western blot analyses, degradation time courses of 11 protein changes, and structural analyses of the PMM2 protein, which suggest that the loss-of-function of most mutant proteins was based on their increased susceptibility to degradation or aggregation compared to the wild type protein, considering PMM2 deficiency as a conformational disease.

Vaes et al. [12] confirmed the results of Vega et al. [31] in their genetic analysis utilizing the NPCRS, showing that variants involving F and stabilization (p.Val231Met, p.Cys241Ser, p.Asp148Asn, p.Arg162Trp, p.Phe183Ser, and p.Ile132Thr in their study) resulted in a significantly lower NPCRS score (*p* = 0.044) in the presence of one nonfunctioning allele and therefore a more milder clinical outcome. Our study used a narrower definition of activity category, utilizing only a subset of the variants used by Vaes et al. [12]. Specifically, our study defined a F category that included p.Val231Met, p.Ile132Thr, and p.Asp148Asn and excluded p.Cys241Ser (demonstrated in other analyses to result in a mild phenotype) and p.Arg162Trp since both were classified as U using the Briso-Montiano et al. [23] method, and, in addition, the p.Phe183Ser variant was not represented in our study. Although the definition of F used in this study included 10 total variants, it encompassed a smaller region of the protein and excluded p.Cys241Ser. As a result, using this more restrictive definition, we showed that our F category resulted in a more severe phenotype based upon differences in the biochemical markers. This highlights the difficulty in making broad conclusions about genotype/phenotype correlations for PMM2-CDG and how the choice of genotypic activity category or grouping for the analysis can result in differing conclusions about the phenotype. It also underscores the value of adding additional phenotypic measures such as biochemical parameters to uncover potential relationships to genotype, particularly the coagulation parameters and liver enzymes.

In addition to activity categories or regions of the PMM2 enzyme predicted to have specific pathogenic mechanisms, there are some individual or single variants that are thought to lead to severe multisystem phenotypes (the most common biallelic variant) and, likewise, there are some variants associated with close to normal IQ [7, 32]. We analyzed previous data [23] to see if disease severity could be determined by the least severe of two *PMM2* variants, such that, when one of the variants exerts a severe effect (e.g., p.Arg141His), the second one could allow for at least partial functioning of the PMM2 allele (Figure 2). In our analysis, severe nonfunctioning variants combined with specific individual variants did show a genotype/phenotype relationship; specifically, p.Cys241Ser resulted in a milder phenotype, while p.Val231Met was more severe, which was in agreement with previous studies [27, 33]. It has been reported that patients with very low PMM2 activity can show a relatively mild phenotype and vice versa [33, 34]. Between patients with the same genotype, differences in disease severity can be seen, although interfamilial clinical homogeneity was generally observed [27, 35, 36].

In the second genotype/phenotype analysis performed that follows Vaes et al. [12], comparing participants with a severe nonfunctioning variant combined with other individual variants and/or activity categories, six patients were excluded because they had two of the nonfunctioning variants, so we sought to further characterize these patients. Not all clinical or biochemical parameters were collected at baseline for these patients (Table S10), so the data is too sparse to draw significant conclusions; however, interestingly, these six patients represent only two different variant pairs, with a common variant, p.Thr237Met; specifically, three patients were p.Arg141His/p.Thr237Met and three patients were p.Phe157Ser/p.Thr237Met. Based upon available data, the p.Arg141His/p.Thr237Met patients were potentially associated with more severe disease since the antithrombin levels for the three patients were relatively low (less than or equal to 0.5), although the NPCRS scores were not significantly different from the average patient.

When we limited our analysis only to the activity category pairs (based upon Briso-Montiano et al. [23]), it was more difficult to find genotype/phenotype correlations. In particular, there were no relationships apparent with the NPCRS sections; however, we did find that coagulation parameters antithrombin and Factor XI were less dysregulated in the pairings F-F and C-NP, again underscoring the value of adding the biochemical information into the analysis as well as the importance of understanding the factors driving coagulopathies, since they were the most consistently correlated to either a variant or activity region of the protein. The conclusion of F-F being associated with a milder phenotype is consistent with the finding that p.Val231Met, within the F category and otherwise identified as a severe phenotype when in combination with a severe nonfunctioning variant, can nevertheless be found in the homozygous state. The conclusion of C-NP being associated with a milder phenotype is at first sight more difficult to interpret since the combination of a catalysis mutation with NP would at first sight be expected to result in more severe disease but, nevertheless, is consistent with previous findings that even patients with very low PMM2 activity can show a relatively mild phenotype [33, 34]. It also underlines that the expression of a specific variant or activity grouping can depend significantly on the variants or activity grouping it is paired with. For example, the activity category pair F-F correlated with improved coagulation parameters, but in combination with the subset of severe nonfunctioning variants, the F category was correlated with worse coagulation parameters.

Additionally, when limiting our analysis to activity categories only, we found that D-NP resulted in a more severe phenotype as measured by the liver enzyme ALT. This is consistent with our finding, and that of Vaes et al. [12], that D in the presence of a severe nonfunctioning variant can lead to worse outcomes as predicted by the NPCRS.

PMM2 deficiency is associated with many other cellular processes and has been shown to be related to alterations of the polyol pathway [6], endoplasmic reticulum (ER) stress [37, 38], and autophagy [38, 39]. These additional metabolic changes might affect phenotypic severity. There is a possibility that the severity is determined by other environmental or genetic factors [40]. It has been demonstrated that nuclear factor-erythroid factor 2-related factor (Nrf2), a master regulator of the antioxidant response and xenobiotic metabolism, plays a critical role in the ER stress response.

Vega et al. [31] suggested that since all patients are functional hemizygous for one missense change and no patient has ever been reported with two copies of an inactivating variant, the phenotype observed should arise from the variants that retain residual activity. In their series, patients with mild phenotypes carried the mutant proteins p.Pro113Leu, p.Thr237Met, and p.Cys241Ser that retained more than 50% residual activity compared to wild-type proteins. The authors suggested that these variants might play a dominant role in the outcome of the disease, conferring a milder phenotype when associated with a severe variant in a compound heterozygous fashion. The authors reported enzymatic activity from cell lines but did not attempt to correlate their results with clinical outcomes in patients. However, Vals et al. [7] demonstrated excellent clinical outcomes in patients with p.Cys241Ser. Our results indicate the presence of the p.Cys241Ser variant has a moderating effect when combined with a nonfunctioning variant, as measured by significantly lower NPCRS scores and significantly higher IGF-1 (Table 9) as well as positive trends in other biochemical markers. This is similar to the findings reported by Vaes et al. [12] for NPCRS scores alone.

### 4.2. New Variants

We identified six variants (out of 60 unique variants), not previously described in PMM2-CDG, comprised of five missense variants (p.Pro20Leu, p.Tyr64Ser, p.Phe68Cys, p.Tyr76His, and p.Arg238His, classified in the U category) and one frameshift (c.696del p.Ala233Argfs∗100, classified in the NP category), presenting with a range of phenotypes from mild to severe. Novel missense variants are usually difficult to classify. However, due to extensive knowledge about *PMM2*, its variations, and functional domains, all novel variants were classified as likely pathogenic per ACMG criteria. The identification of these variants is important for the correct diagnosis and counseling of parents and prospective parents and is common with the increasing awareness and attention paid by researchers to respective rare genetic diseases such as PMM2-CDG.

### 4.3. Diagnostic and Biologic Relevance

Although we found some differences between activity category pairs and a limited number of biochemical parameters (antithrombin, Factor XI, and ALT) (Table 6(c)), the genotype/phenotype correlations were clearer when looking at activity categories and/or variants combined with a group of severe nonfunctioning variants. Specifically, there was a moderating effect of p.Cys241 on NPCRS clinical and biochemical parameters and an exacerbating effect of p.Val231Met and F on biochemical parameters when combined with severe nonfunctioning variants (Tables 8, 9, and 10).

It is important to find new ways to predict genotype/phenotype correlations. One option is a computational analysis of the structural perturbation of several PMM2 disease-associated variants. This method may be used to complement other in silico methods that assess pathogenicity [41]. It has been demonstrated that molecular dynamic studies are able to pinpoint the main effects on conformation and dynamics, even a case of the relatively mild (p.Glu197Ala) variant in *PMM2*. These computational simulations have the potential to lead to a better understanding of target biological molecules; however, the difficulty of defining genotypes into broad activity categories is underscored by the differing conclusions of severity drawn by Vaes et al. [12] for the F and stabilization category as compared to the narrower category of F used in the current analysis.

### 4.4. Clinical Implications

The lack of broad phenotype/genotype correlations in PMM2 patients limits the information offered to families of a newly diagnosed patient, which affects their empowerment and mental representation of the disease process. Moreover, the clinical domains primarily affected in our series (communication, self-care, mobility, development, and ataxia) have recently been highlighted as crucial factors affecting the quality of life of these patients; they serve as truly patient reported outcome measures (PROM) [42]. This current analysis shows that some specific phenotype/genotype correlations emerge if larger datasets are analyzed through a different lens and by augmenting the clinical parameters with additional biochemical measurements and, thereby, can potentially offer some additional assistance to the affected families.

## 5. Conclusion

Our findings suggest that in a large cohort (*n* = 137) of patients with PMM2-CDG, genotype/phenotype correlations can be made through using different approaches to categorizing the genotype and by utilizing a more thorough description of the phenotype. In particular, a classification that includes a specific subset of nonfunctioning variants in combination with specific variants and/or activity categories shows that additional genotype/phenotype correlations can be made. Specifically, the analysis suggests the p.Cys241Ser variant mitigated the severity of the expression of PMM2-CDG, while p.Val231Met, F, or D variants were associated with more severe expression of PMM2-CDG.

## 6. Web Resources

The following are the web resources used in this study: SAS(R) Version 9.4 (http://www.sas.com), the Genome Aggregation Database (gnomAD) (https://gnomad.broadinstitute.org/), and ClinVar (https://www.ncbi.nlm.nih.gov/clinvar/).

## Figures and Tables

**Table 1:** Summary of demographics.

	**All (** **N** ** = 137)**	**First analysis ** **population** ^a^ ** (** **N** ** = 88)**	**Second analysis ** **population** ^b^ ** (** **N** ** = 96)**
Age (years)			
*n*	137	88	96
Median	10.40	10.00	9.50
Minimum, maximum	0.5, 68.4	0.5, 68.4	0.5, 48.6
Age group (years)			
0–<6 years	44 (32.1%)	31 (35.2%)	35 (36.5%)
6–<12 years	32 (23.4%)	18 (20.5%)	20 (20.8%)
12–<18 years	19 (13.9%)	10 (11.4%)	13 (13.5%)
≥18 years	42 (30.7%)	29 (33.0%)	28 (29.2%)
Sex			
Female	64 (46.7%)	36 (40.9%)	44 (45.8%)
Male	73 (53.3%)	52 (59.1%)	52 (54.2%)

^a^First analysis population includes all participants whose variants are in an activity category with at least four participants, with no variants in the uncertain category.

^b^Participants with only one of the nonfunctioning variants: p.Arg141His, p.Phe157Ser, p.Asp188Gly, p.Thr237Met, or p.Thr237Arg.

**Table 2:** Participants with pathogenic variants: NPCRS scores.

	**All (** **N** = 137**)**	**First analysis ** **population** ^a^ ** (** **N** ** = 88)**	**Second analysis ** **population** ^b^ ** (** **N** ** = 96)**
Section 1 subscore			
*n*	117	82	86
Mean (SD)	8.53 (3.832)	9.35 (3.772)	8.95 (4.070)
Median	9.00	9.00	9.00
Min, max	0.0, 19.0	2.0, 19.0	0.0, 19.0
Section 2 subscore			
*n*	117	82	86
Mean (SD)	4.18 (2.824)	4.60 (2.605)	4.29 (2.946)
Median	4.00	4.00	4.00
Min, max	0.0, 17.0	0.0, 14.0	0.0, 17.0
Section 3 subscore			
*n*	115	81	85
Mean (SD)	11.61 (3.776)	12.44 (3.543)	11.91 (3.816)
Median	11.00	12.00	12.00
Min, max	2.0, 21.0	4.0, 21.0	4.0, 21.0
Total score			
*n*	117	82	86
Mean (SD)	24.12 (8.316)	26.24 (7.664)	25.01 (8.632)
Median	23.00	25.00	24.00
Min, max	2.0, 46.0	8.0, 46.0	5.0, 46.0

Abbreviations: Max = maximum, Min = minimum, SD = standard deviation.

^a^First analysis population includes all participants whose variants are in an activity category with at least four participants, with no variants in the uncertain category.

^b^Participants with only one of the nonfunctioning variants: p.Arg141His, p.Phe157Ser, p.Asp188Gly, p.Thr237Met, or p.Thr237Arg.

**Table 3:** Participants with pathogenic variants: laboratory parameters.

	**All (** **N** ** = 137)**	**First analysis ** **population** ^a^ ** (** **N** ** = 88)**	**Second analysis ** **population** ^b^ ** (** **N** ** = 96)**
Antithrombin activity			
*n*	117	79	83
Mean (SD)	0.57 (0.257)	0.53 (0.249)	0.55 (0.244)
Median	0.52	0.44	0.46
Min, max	0.2, 1.3	0.2, 1.2	0.2, 1.2
≤ LLN, *n* (%)	93 (79.5%)	66 (83.5%)	68 (81.9%)
≥ ULN, *n* (%)	2 (1.7%)	1 (1.3%)	0
Protein C activity (%)			
*n*	95	66	66
Mean (SD)	62.32 (27.751)	59.62 (28.804)	61.64 (28.338)
Median	60.00	58.00	59.00
Min, max	8.0, 166.0	8.0, 166.0	8.0, 166.0
≤ LLN, *n* (%)	54 (56.8%)	42 (63.6%)	41 (62.1%)
≥ ULN, *n* (%)	1 (1.1%)	1 (1.5%)	1 (1.5%)
Factor XI activity (%)			
*n*	114	79	82
Mean (SD)	60.03 (27.353)	55.41 (25.736)	57.05 (26.627)
Median	54.50	52.00	52.00
Min, max	11.0, 124.0	11.0, 121.4	11.0, 124.0
≤ LLN, *n* (%)	64 (56.1%)	50 (63.3%)	52 (63.4%)
≥ ULN, *n* (%)	0	0	0
Factor IX (%)			
*n*	104	71	73
Mean (SD)	86.23 (28.374)	82.95 (26.862)	84.30 (29.175)
Median	84.35	83.00	82.00
Min, max	16.0, 197.0	16.0, 194.0	16.0, 197.0
≤ LLN, *n* (%)	11 (10.6%)	10 (14.1%)	8 (11.0%)
≥ ULN, *n* (%)	5 (4.8%)	2 (2.8%)	3 (4.1%)

Abbreviations: LLN = lower limit of normal, Max = maximum, Min = minimum, SD = standard deviation, ULN = upper limit of normal.

^a^First analysis population includes all participants whose variants are in an activity category with at least four participants, with no variants in the uncertain category.

^b^Participants with only one of the nonfunctioning variants: p.Arg141His, p.Phe157Ser, p.Asp188Gly, p.Thr237Met, or p.Thr237Arg.

**Table 4:** Most frequent pathogenic variants.

**Short name**	**HGVS protein ** **(NP_000294.1)**	**HGVS nucleotide ** **(NP_000303.3)**	**Category**	**All (** **N** ** = 137)**	**First analysis ** **population** ^a^ ** (** **N** ** = 88)**	**Second analysis ** **population** ^b^ ** (** **N** ** = 96)**
R141H	p.Arg141His	c.422G>A	Catalytic	80 (58.4%)	67 (76.1%)	77 (80.2%)
P113L	p.Pro113Leu	c.338C>T	Dimerization	29 (21.2%)	24 (27.3%)	19 (19.8%)
F119L	p.Phe119Leu	c.357C>A	Dimerization	17 (12.4%)	12 (13.6%)	12 (12.5%)
V231M	p.Val231Met	c.691G>A	Folding	11 (8.0%)	7 (8.0%)	10 (10.4%)
C241S	p.Cys241Ser	c.722G>C	Uncertain	11 (8.0%)	—	8 (8.3%)
F157S	p.Phe157Ser	c.470 T>C	Folding w/o activity	9 (6.6%)	1 (1.1%)	6 (6.3%)
R162W	p.Arg162Trp	c.484C>T	Uncertain	9 (6.6%)	—	3 (3.1%)
T237M	p.Thr237Met	c.710C>T	Folding w/o activity	9 (6.6%)	4 (4.5%)	3 (3.1%)
T237R	p.Thr237Arg	c.710C>G	Folding w/o activity	8 (5.8%)	5 (5.7%)	8 (8.3%)
V129M	p.Val129Met	c.385G>A	Folding	7 (5.1%)	6 (6.8%)	6 (6.3%)

*Note:* There are 60 unique variants identified in the study.

^a^First analysis population includes all participants whose variants are in an activity category with at least four participants, with no variants in the uncertain category.

^b^Participants with only one of the nonfunctioning variants: p.Arg141His, p.Phe157Ser, p.Asp188Gly, p.Thr237Met, or p.Thr237Arg.

**Table 5:** Pathogenic variant categories (*N* = 137).

Catalytic/activation: *n* = 98 (71.5%)		
p.Arg141His (R141H) *n* = 80 (58.4%)	p.Arg123Gln (R123Q) *n* = 5 (3.6%)	p.Asn216Ile (N216I) *n* = 4 (2.9%)
p.Gly15Ala (G15A) *n* = 3 (2.2%)	p.Arg21Gly (R21G) *n* = 2 (1.5%)	p.Thr18Ser (T18S) *n* = 1 (0.7%)
p.Gln177His (Q177H) *n* = 1 (0.7%)	p.Gly208Ala (G208A) *n* = 1 (0.7%)	p.Asn216Ser (N216S) *n* = 1 (0.7%)
Dimerization: *n* = 49 (35.8%)		
p.Pro113Leu (P113L) *n* = 29 (21.2%)	p.Phe119Leu (F119L) *n* = 17 (12.4%)	p.Ala108Val (A108V) *n* = 3 (2.2%)
Folding: *n* = 47 (34.3%)		
p.Val231Met (V231M) *n* = 11 (8.0%)	p.Val129Met (V129M) *n* = 7 (5.1%)	p.Asp148Asn (D148N) *n* = 4 (2.9%)
p.Gly214Ser (G214S) *n* = 4 (2.9%)	p.Ile132Thr (I132T) *n* = 3 (2.2%)	p.Val44Ala (V44A) *n* = 2 (1.5%)
p.Lys51Arg (K51R) *n* = 2 (1.5%)	p.Ile153Thr (I153T) *n* = 2 (1.5%)	p.Phe207Ser (F207S) *n* = 2 (1.5%)
p.Asp223Asn (D223N) *n* = 2 (1.5%)	p.Cys9Tyr (C9Y) *n* = 1 (0.7%)	p.Leu32Arg (L32R) *n* = 1 (0.7%)
p.Gln33Pro (Q33P) *n* = 1 (0.7%)	p.Gly57Arg (G57R) *n* = 1 (0.7%)	p.Asp65Tyr (D65Y) *n* = 1 (0.7%)
p.Pro69Ser (P69S) *n* = 1 (0.7%)	p.Thr226Ser (T226S) *n* = 1 (0.7%)	p.Tyr229Ser (Y229S) *n* = 1 (0.7%)
Uncertain: *n* = 32 (23.4%)		
p.Cys241Ser (C241S) *n* = 11 (8.0%)	p.Arg162Trp (R162W) *n* = 9 (6.6%)	p.Ser47Leu (S47L) *n* = 2 (1.5%)
p.Pro20Leu (P20L) *n* = 1 (0.7%)	p.Tyr64Ser (Y64S) *n* = 1 (0.7%)	p.Phe68Cys (F68C) *n* = 1 (0.7%)
p.Tyr76His (Y76H) *n* = 1 (0.7%)	p.Pro113Ser (P113S) *n* = 1 (0.7%)	p.Pro113Thr (P113T) *n* = 1 (0.7%)
p.Thr118Ser (T118S) *n* = 1 (0.7%)	p.Met126Thr (M126T) *n* = 1 (0.7%)	p.Arg238His (R238H) *n* = 1 (0.7%)
c.639-1G>T *n* = 1 (0.7%)		
Folding w/o activity: *n* = 26 (19.0%)		
p.Phe157Ser (F157S) *n* = 9 (6.6%)	p.Thr237Met (T237M) *n* = 9 (6.6%)	p.Thr237Arg (T237R) *n* = 8 (5.8%)
No protein: *n* = 17 (12.4%)		
p.Glu139Lys (E139K) *n* = 4 (2.9%)	p.Cys9AlafsTer27 (C9fs) *n* = 4 (2.9%)	c.640-23A>G *n* = 2 (1.5%)
p.Gln37Ter (Q37X) *n* = 1 (0.7%)	p.Arg123Ter (R123X) *n* = 1 (0.7%)	c.178+3A>T (IVS2+3A > T) *n* = 1 (0.7%)
15 KB DELETION 16p13.2 *n* = 1 (0.7%)	c.523+3A>G *n* = 1 (0.7%)	p.Ala233Argfs∗100 (696delA) *n* = 1 (0.7%)
p.Glu151IlefsTer2 (451-454DELGAAA) *n* = 1 (0.7%)		
Linker-2: *n* = 6 (4.4%)		
p.Phe183Ser (F183S) *n* = 2 (1.5%)	p.Asp188Gly (D188G) *n* = 2 (1.5%)	p.Pro184Thr (P184T) *n* = 1 (0.7%)
p.Gly186Arg (G186R) *n* = 1 (0.7%)		

**(a):** 

	**Statistic**	**C-C (** **N** ** = 8)**	**C-D (** **N** ** = 28)**	**C-F (** **N** ** = 26)**	**C-FWO (** **N** ** = 4)**	**C-NP (** **N** ** = 6)**	**D-FWO (** **N** ** = 6)**	**D-NP (** **N** ** = 5)**	**F-F (** **N** ** = 5)**
Age (years)	*n*	8	28	26	4	6	6	5	5
	Mean (SD)	18.50 (19.914)	15.11 (14.637)	13.19 (11.993)	15.00 (10.708)	22.67 (23.864)	7.83 (2.483)	7.60 (7.403)	22.40 (14.502)
	*p* value^a^	0.462							

Sex	Female, *n* (%)	3 (37.5%)	12 (42.9%)	10 (38.5%)	2 (50.0%)	3 (50.0%)	2 (33.3%)	2 (40.0%)	2 (40.0%)
	Male, *n* (%)	5 (62.5%)	16 (57.1%)	16 (61.5%)	2 (50.0%)	3 (50.0%)	4 (66.7%)	3 (60.0%)	3 (60.0%)
	*p* value^a^	0.9719							

^a^For age, *p* value is from an analysis of variance. For sex, *p* value is from a chi-squared test.

**(b):** 

	**Statistic**	**C-C (** **N** ** = 8)**	**C-D (** **N** ** = 28)**	**C-F (** **N** ** = 26)**	**C-FWO (** **N** ** = 4)**	**C-NP (** **N** ** = 6)**	**D-FWO (** **N** ** = 6)**	**D-NP (** **N** ** = 5)**	**F-F (** **N** ** = 5)**
Section 1 subscore	*n*	7	25	25	4	5	6	5	5
	Mean (SD)	5.9 (3.44)	5.7 (2.82)	5.0 (2.96)	3.8 (0.50)	6.4 (0.89)	6.5 (2.43)	4.4 (1.52)	4.4 (1.82)
	*p* value^a^	0.4563							

Section 2 subscore	*n*	7	25	25	4	5	6	5	5
	Mean (SD)	5.3 (4.03)	4.4 (2.77)	4.5 (2.24)	3.5 (1.91)	5.2 (2.17)	4.0 (2.28)	7.0 (2.35)	4.0 (2.55)
	*p* value^a^	0.4563							

Section 3 subscore	*n*	7	24	25	4	5	6	5	5
	Mean (SD)	12.6 (3.64)	12.8 (2.98)	11.6 (4.11)	11.5 (2.38)	15.0 (2.35)	14.7 (3.56)	13.4 (2.88)	9.8 (3.56)
	*p* value^a^	0.4563							

Total score	*n*	7	25	25	4	5	6	5	5
	Mean (SD)	23.7 (8.30)	22.3 (6.72)	21.0 (7.46)	18.8 (3.30)	26.6 (5.13)	25.2 (6.94)	24.8 (4.44)	18.2 (4.09)
	*p* value^a^	0.4563							

^a^ *p* values are the FDR adjusted *p* values calculated using the Kruskal–Wallis test to globally compare the groups.

**(c):** 

	**Statistic**	**C-C (** **N** ** = 8)**	**C-D (** **N** ** = 28)**	**C-F (** **N** ** = 26)**	**C-FWO (** **N** ** = 4)**	**C-NP (** **N** ** = 6)**	**D-FWO (** **N** ** = 6)**	**D-NP (** **N** ** = 5)**	**F-F (** **N** ** = 5)**
Antithrombin activity	*n*	7	22	25	4	6	6	5	4
	Mean (SD)	0.56 (0.235)	0.50 (0.213)	0.46 (0.222)	0.48 (0.220)	0.91 (0.191)	0.54 (0.187)	0.37 (0.159)	0.79 (0.350)
	*p* value^a^	0.0541							

Protein C activity (%)	*n*	4	18	22	4	5	5	5	3
	Mean (SD)	64.00 (30.408)	55.17 (21.302)	60.64 (35.792)	51.00 (28.343)	89.40 (18.995)	58.00 (10.124)	35.20 (10.378)	78.33 (36.116)
	*p* value^a^	0.1538							

Factor XI activity (%)	*n*	7	22	24	4	6	6	5	5
	Mean (SD)	57.73 (27.412)	50.74 (19.826)	45.91 (20.121)	46.50 (17.635)	96.00 (17.239)	68.57 (30.937)	38.60 (19.527)	77.82 (27.429)
	*p* value^a^	0.0384							

Factor IX (%)	*n*	7	19	21	4	5	6	5	4
	Mean (SD)	92.39 (17.216)	80.74 (34.639)	81.36 (23.368)	80.50 (18.212)	91.00 (26.077)	90.05 (31.559)	63.00 (22.989)	91.95 (23.594)
	*p* value^a^	0.4544							

A-oligo/di-oligo transferrin ratio	*n*	5	8	16	1	4	3	1	4
	Mean (SD)	0.16 (0.116)	0.19 (0.187)	0.27 (0.243)	0.18 (.)	0.01 (0.009)	0.11 (0.134)	0.54 (.)	0.09 (0.094)
	*p* value^a^	0.1296							

Mono-oligo/di-oligo transferrin ratio	*n*	5	8	16	1	4	3	1	4
	Mean (SD)	0.63 (0.285)	0.63 (0.399)	0.79 (0.681)	0.49 (.)	0.08 (0.042)	0.55 (0.499)	2.34 (.)	0.34 (0.269)
	*p* value^a^	0.1296							

^a^ *p* values are the FDR adjusted *p* values calculated using the Kruskal–Wallis test to globally compare the groups.

**(d):** 

	**Statistic**	**C-NP (** **N** ** = 6)**	**F-F (** **N** ** = 5)**	**Other (** **N** ** = 77)**
Antithrombin activity	*n*	6	4	69
	Mean (SE)	0.91 (0.089)	0.79 (0.108)	0.49 (0.026)
	95% CI for the mean	0.73, 1.08	0.57, 1.01	0.43, 0.54
	LS mean difference^a^ (SE)	0.42 (0.092)	0.30 (0.112)	
	95% CI for the LS mean difference	0.24, 0.60	0.08, 0.53	
	*p* value^a^	<0.0001	0.0081	

Factor XI activity (%)	*n*	6	5	68
	Mean (SE)	96.00 (9.058)	77.82 (9.922)	50.19 (2.690)
	95% CI for the mean	77.96, 114.04	58.06, 97.58	44.83, 55.54
	LS mean difference^a^ (SE)	45.81 (9.449)	27.63 (10.280)	
	95% CI for the LS mean difference	27.00, 64.63	7.16, 48.11	
	*p* value^a^	<0.0001	0.0088	

Abbreviations: LS = least square, SE = standard error, SD = standard deviation.

^a^Least square means differences and *p* values are for the comparison against the other variant categories combined (other column) using an ANOVA model with variant category as the independent variable.

**(e):** 

	**Statistic**	**D-NP (** **N** ** = 5)**	**Other (** **N** ** = 77)**
Alanine transaminase (IU/L)	*n*	5	82
	Mean (SE)	192.40 (33.834)	55.36 (8.355)
	95% CI for the mean	125.13, 259.67	38.75, 71.97
	LS mean difference^a^ (SE)	137.039 (34.8504)	
	95% CI for the LS mean difference	67.75, 206.33	
	*p* value^a^	0.0002	

Abbreviations: LS = least square, SE = standard error, SD = standard deviation.

^a^Least square means differences and *p* values are for the comparison against the other variant categories combined (other column) using an ANOVA model with variant category as the independent variable.

**Table 7:** Effect of dimerization variants for participants with a nonfunctioning variant. Second analysis population summary of demographics by analysis group.

	**Group 1** ^a^ ** (** **N** = 34**)**		**Group 2** ^b^ ** (** **N** = 62**)**		**p** ** value** ^c^
	**n**	**Mean (SD)**	**n**	**Mean (SD)**	
Age (years)	34	13.82 (13.570)	62	13.42 (12.047)	0.8809
Male	14	41.2%	30	48.4%	—
Female	20	58.8%	32	51.6%	0.8809
NPCRS section 1 subscore	31	5.9 (2.73)	55	4.6 (2.83)	0.0798
NPCRS section 2 subscore	31	4.3 (2.65)	55	4.3 (3.13)	0.8099
NPCRS section 3 subscore	30	13.1 (3.14)	55	11.2 (4.01)	0.0763
NPCRS total score	31	22.9 (6.74)	55	20.1 (7.98)	0.0984
Antithrombin activity	28	0.51 (0.205)	55	0.57 (0.262)	0.8754
Protein C activity (%)	23	55.78 (19.254)	43	64.77 (31.934)	0.8754
Factor XI activity (%)	28	54.56 (23.205)	54	58.35 (28.359)	0.8754
Factor IX (%)	25	82.98 (33.524)	48	84.99 (26.993)	0.8754
A-oligo/di-oligo transferrin ratio	11	0.17 (0.171)	30	0.20 (0.204)	0.8754
Mono-oligo/di-oligo transferrin ratio	11	0.61 (0.403)	30	0.61 (0.565)	0.8754
Insulin-like growth factor 1 (ng/mL)	26	103.35 (62.010)	46	148.98 (98.364)	0.2853
Insulin-like growth factor binding protein 3 (*μ*g/L)	13	13067.08 (14234.733)	27	18232.21 (21362.155)	0.8754
Alkaline phosphatase (IU/L)	30	180.23 (90.160)	54	186.03 (102.950)	0.9330
Alanine transaminase (IU/L)	34	60.34 (64.797)	59	52.16 (53.839)	0.8754
Aspartate aminotransferase (IU/L)	32	53.99 (44.144)	57	59.29 (73.903)	0.8754
Ceruloplasmin (*μ*mol/L)	20	2.15 (0.609)	38	1.83 (0.520)	0.2853

^a^Group 1 comprises participants with one nonfunctioning variant combined with a variant in the dimerization domain.

^b^Group 2 comprises participants with one nonfunctioning variant and any other variant.

^c^ *p* values are the FDR adjusted *p* values calculated using the Kruskal–Wallis test to compare Group 1 and Group 2.

**Table 8:** Effect of folding variant for participants with a nonfunctioning variant second analysis population summary of demographics by analysis group.

	**Group 1** ^a^ ** (** **N** = 29**)**		**Group 2** ^b^ ** (** **N** = 67**)**		**p** ** value** ^c^
	**n**	**Mean (SD)**	**n**	**Mean (SD)**	
Age (years)	29	12.28 (11.689)	67	14.12 (12.933)	0.5112
Female	14	48.3%	30	44.8%	—
Male	15	51.7%	37	55.2%	0.7533
NPCRS section 1 subscore	27	5.1 (2.74)	59	5.0 (2.92)	0.8668
NPCRS section 2 subscore	27	4.8 (3.32)	59	4.1 (2.76)	0.8360
NPCRS section 3 subscore	27	11.5 (3.96)	58	12.1 (3.77)	0.8360
NPCRS total score	27	21.5 (7.44)	59	20.9 (7.78)	0.8668
Antithrombin activity	27	0.44 (0.200)	56	0.60 (0.250)	0.0316⁣^∗^
Protein C activity (%)	23	57.61 (36.400)	43	63.79 (23.133)	0.1240
Factor XI activity (%)	26	44.63 (20.237)	56	62.82 (27.406)	0.0316⁣^∗^
Factor IX (%)	23	80.61 (24.318)	50	85.99 (31.239)	0.8539
A-oligo/di-oligo transferrin ratio	17	0.27 (0.234)	24	0.13 (0.139)	0.0785
Mono-oligo/di-oligo transferrin ratio	17	0.79 (0.653)	24	0.49 (0.370)	0.1240
Insulin-like growth factor 1 (ng/mL)	23	118.99 (87.661)	49	138.84 (90.241)	0.4815
Insulin-like growth factor binding protein 3 (*μ*g/L)	11	16073.36 (18842.579)	29	16735.68 (19799.525)	0.8539
Alkaline phosphatase (IU/L)	25	209.67 (136.193)	59	173.07 (75.364)	0.4815
Alanine transaminase (IU/L)	27	70.07 (66.138)	66	49.05 (53.482)	0.0364⁣^∗^
Aspartate aminotransferase (IU/L)	27	84.33 (100.620)	62	45.65 (35.171)	0.1853
Ceruloplasmin (*μ*mol/L)	18	1.73 (0.461)	40	2.04 (0.590)	0.1240

^a^Group 1 comprises participants with one nonfunctioning variant combined with a variant in the folding domain.

^b^Group 2 comprises participants with one nonfunctioning variant and any other variant.

^c^ *p* values are the FDR adjusted *p* values calculated using the Kruskal–Wallis test to compare Group 1 and Group 2.

⁣^∗^*p* < 0.05.

**Table 9:** Effect of p.Cys241Ser for participants with a nonfunctioning variant second analysis population summary of demographics by analysis group.

	**Group** ^a^ ** (** **N** ** = 8)**		**Group 2** ^b^ ** (** **N** ** = 88)**		**p** ** value** ^c^
	**n**	**Mean (SD)**	**n**	**Mean (SD)**	
Age (years)	8	18.63 (7.596)	88	13.10 (12.823)	0.2349
Female	5	62.5%	39	44.3%	—
Male	3	37.5%	49	55.7%	0.3256
NPCRS section 1 subscore	6	1.2 (2.04)	80	5.4 (2.69)	0.0024⁣^∗^
NPCRS section 2 subscore	6	2.5 (1.97)	80	4.4 (2.97)	0.0684
NPCRS section 3 subscore	6	7.5 (1.64)	79	12.2 (3.73)	0.0028⁣^∗^
NPCRS total score	6	11.2 (4.54)	80	21.9 (7.30)	0.0024⁣^∗^
Antithrombin activity	8	0.75 (0.215)	75	0.53 (0.238)	0.0811
Protein C activity (%)	6	83.50 (24.378)	60	59.45 (27.944)	0.0884
Factor XI activity (%)	8	79.35 (33.194)	74	54.64 (24.913)	0.0884
Factor IX (%)	8	91.51 (18.710)	65	83.41 (30.199)	0.2517
A-oligo/di-oligo transferrin ratio	2	0.02 (0.019)	39	0.20 (0.195)	0.0969
Mono-oligo/di-oligo transferrin ratio	2	0.12 (0.106)	39	0.64 (0.522)	0.0969
Insulin-like growth factor 1 (ng/mL)	6	254.93 (75.173)	66	121.37 (82.251)	0.0235⁣^∗^
Insulin-like growth factor binding protein 3 (*μ*g/L)	5	25696.00 (30048.020)	35	15247.48 (17524.235)	0.3037
Alkaline phosphatase (IU/L)	8	135.13 (45.992)	76	189.10 (100.812)	0.1451
Alanine transaminase (IU/L)	8	30.75 (23.891)	85	57.45 (59.654)	0.1471
Aspartate aminotransferase (IU/L)	8	29.13 (11.922)	81	60.18 (66.972)	0.0884
Ceruloplasmin (*μ*mol/L)	4	2.19 (0.742)	54	1.92 (0.557)	0.4802

^a^Group 1 comprises participants with one nonfunctioning variant combined with the p.Cys241Ser variant.

^b^Group 2 comprises participants with one nonfunctioning variant and any other variant.

^c^ *p* values are the FDR adjusted *p* values calculated using the Kruskal–Wallis test to compare Group 1 and Group 2.

⁣^∗^*p* < 0.05.

**Table 10:** Effect of p.Val231Met for participants with a nonfunctioning variant second analysis population summary of demographics by analysis group.

	**Group 1** ^a^ **(** **N** = 10**)**		**Group 2** ^b^ ** (** **N** = 86**)**		**p** ** value** ^c^
	**n**	**Mean (SD)**	**n**	**Mean (SD)**	
Age (years)	10	6.40 (7.043)	86	14.40 (12.795)	0.0559
Female	6	60.0%	38	44.2%	—
Male	4	40.0%	48	55.8%	0.3447
NPCRS section 1 subscore	9	6.2 (3.42)	77	4.9 (2.77)	0.2684
NPCRS section 2 subscore	9	6.8 (4.35)	77	4.0 (2.63)	0.0842
NPCRS section 3 subscore	9	13.0 (4.12)	76	11.8 (3.79)	0.4561
NPCRS total score	9	26.0 (7.57)	77	20.5 (7.48)	0.0852
Antithrombin activity	10	0.41 (0.160)	73	0.57 (0.248)	0.1177
Protein C activity (%)	8	36.75 (17.726)	58	65.07 (27.897)	0.0260⁣^∗^
Factor XI activity (%)	10	38.84 (22.276)	72	59.58 (26.320)	0.0291⁣^∗^
Factor IX (%)	9	72.41 (27.081)	64	85.97 (29.268)	0.4873
A-oligo/di-oligo transferrin ratio	7	0.26 (0.238)	34	0.18 (0.185)	0.4778
Mono-oligo/di-oligo transferrin ratio	7	0.81 (0.666)	34	0.57 (0.489)	0.4478
Insulin-like growth factor 1 (ng/mL)	9	96.88 (76.556)	63	137.59 (90.355)	0.3943
Insulin-like growth factor binding protein 3 (*μ*g/L)	6	10658.50 (13657.366)	34	17593.84 (20115.271)	0.6224
Alkaline phosphatase (IU/L)	10	294.94 (165.770)	74	168.96 (75.034)	0.0260⁣^∗^
Alanine transaminase (IU/L)	10	110.38 (86.038)	83	48.50 (50.283)	0.0199⁣^∗^
Aspartate aminotransferase (IU/L)	10	141.00 (139.807)	79	46.80 (37.920)	0.0410⁣^∗^
Ceruloplasmin (*μ*mol/L)	8	1.77 (0.483)	50	1.97 (0.580)	0.5597

^a^Group 1 comprises participants with one nonfunctioning variant combined with the p.Val231Met variant.

^b^Group 2 comprises participants with one nonfunctioning variant and any other variant.

^c^ *p* values are the FDR adjusted *p* values calculated using the Kruskal–Wallis test to compare Group 1 and Group 2.

⁣^∗^*p* < 0.05.

## Data Availability

The data that support the findings of this study are available from the corresponding author upon reasonable request.
