# Supplementary material for: Genotype/Phenotype Relationship: Lessons From 137 Patients With PMM2-CDG
Source: Hum Mutat. 2024 Oct 3;2024:8813121. doi: 10.1155/2024/8813121 (PMC11922042; doi:10.1155/2024/8813121)
Supplement: Supporting Information — Additional supporting information can be found online in the Supporting Information section. [file 8813121.f1.docx]

**Supplementary Materials**

**Supp. Table S1. Demographics by Country (N=137)**

|  | Belgium (N=13) | Czech Republic (N=17) | France (N=19) | Italy (N=8) | Poland (N=9) | Portugal (N=7) | Spain (N=8) | The Netherlands (N=10) | US (N=46) |
| --- | --- | --- | --- | --- | --- | --- | --- | --- | --- |
| Age (years) | | | | | | | | | |
| n | 13 | 17 | 19 | 8 | 9 | 7 | 8 | 10 | 46 |
| Median | 18.10 | 10.30 | 14.80 | 34.15 | 5.00 | 9.30 | 13.80 | 26.30 | 6.60 |
| Minimum, Maximum | 0.9, 43.3 | 1.6, 31.4 | 3.5, 31.7 | 6.8, 48.6 | 0.5, 17.1 | 5.4, 12.6 | 3.7, 20.5 | 4.8, 46.7 | 0.7, 68.4 |
| Age Group (years) | | | | | | | | | |
| 0 - <6 years | 4 (30.8%) | 5 (29.4%) | 2 (10.5%) | 0 | 6 (66.7%) | 1 (14.3%) | 1 (12.5%) | 3 (30.0%) | 22 (47.8%) |
| 6 - <12 years | 2 (15.4%) | 4 (23.5%) | 5 (26.3%) | 1 (12.5%) | 1 (11.1%) | 5 (71.4%) | 1 (12.5%) | 1 (10.0%) | 12 (26.1%) |
| 12 - <18 years | 0 | 3 (17.6%) | 5 (26.3%) | 1 (12.5%) | 2 (22.2%) | 1 (14.3%) | 5 (62.5%) | 0 | 2 (4.3%) |
| >= 18 years | 7 (53.8%) | 5 (29.4%) | 7 (36.8%) | 6 (75.0%) | 0 | 0 | 1 (12.5%) | 6 (60.0%) | 10 (21.7%) |
| Sex | | | | | | | | | |
| Female | 8 (61.5%) | 9 (52.9%) | 14 (73.7%) | 1 (12.5%) | 4 (44.4%) | 4 (57.1%) | 5 (62.5%) | 5 (50.0%) | 14 (30.4%) |
| Male | 5 (38.5%) | 8 (47.1%) | 5 (26.3%) | 7 (87.5%) | 5 (55.6%) | 3 (42.9%) | 3 (37.5%) | 5 (50.0%) | 32 (69.6%) |

**Supp. Table S2. Demographics by Region**

|  | All (N=137) | EU (N=91) | US (N=46) |
| --- | --- | --- | --- |
| Age (years) | | | |
| n | 137 | 91 | 46 |
| Median | 10.40 | 12.80 | 6.60 |
| Minimum, Maximum | 0.5, 68.4 | 0.5, 48.6 | 0.7, 68.4 |
| Age Group (years) | | | |
| 0 - <6 years | 44 (32.1%) | 22 (24.2%) | 22 (47.8%) |
| 6 - <12 years | 32 (23.4%) | 20 (22.0%) | 12 (26.1%) |
| 12 - <18 years | 19 (13.9%) | 17 (18.7%) | 2 (4.3%) |
| >= 18 years | 42 (30.7%) | 32 (35.2%) | 10 (21.7%) |
| Sex | | | |
| Female | 64 (46.7%) | 50 (54.9%) | 14 (30.4%) |
| Male | 73 (53.3%) | 41 (45.1%) | 32 (69.6%) |
| The difference in age distribution between the EU and the US is statistically significant (p-value = 0.0038 when comparing medians, p-value = 0.0079 when comparing age groups). | | | |

**Supp. Table S3. Variant Pair Activity Categories (First Analysis)**

|  | All Participants (N=137) |
| --- | --- |
| Included in First Analysis† (N=88) | |
| Catalytic - Dimerization | 28 (20.4%) |
| Catalytic - Folding | 26 (19.0%) |
| Catalytic - Catalytic | 8 (5.8%) |
| Catalytic - No Protein | 6 (4.4%) |
| Dimerization - Folding w/o Activity | 6 (4.4%) |
| Dimerization - No Protein | 5 (3.6%) |
| Folding - Folding | 5 (3.6%) |
| Catalytic - Folding w/o Activity | 4 (2.9%) |
| Excluded from First Analysis (N=49) | |
| Catalytic - Uncertain | 15 (10.9%) |
| Folding w/o Activity - Uncertain | 6 (4.4%) |
| No Protein - Uncertain | 4 (2.9%) |
| Catalytic - Linker-2 | 3 (2.2%) |
| Dimerization - Dimerization | 3 (2.2%) |
| Folding - Folding w/o Activity | 3 (2.2%) |
| Folding - Uncertain | 3 (2.2%) |
| Folding w/o Activity - Folding w/o Activity | 3 (2.2%) |
| Dimerization - Folding | 2 (1.5%) |
| Dimerization - Uncertain | 2 (1.5%) |
| Folding - Linker-2 | 2 (1.5%) |
| Folding - No Protein | 1 (0.7%) |
| Folding w/o Activity - No Protein | 1 (0.7%) |
| Linker-2 - Uncertain | 1 (0.7%) |
| † First Analysis Population includes all participants whose variants are in an activity category with at least 4 participants, with no variants in the uncertain category. | |

**Supp. Table S4. Variant Pair Activity Categories (Second Analysis)**

|  | All Participants (N=137) |
| --- | --- |
| Excluded in Second Analysis: Participants with zero or two non-functioning allele (N=41) | |
| Catalytic - Uncertain | 5 (3.6%) |
| Dimerization - No Protein | 5 (3.6%) |
| Folding - Folding | 5 (3.6%) |
| No Protein - Uncertain | 4 (2.9%) |
| Catalytic - Folding w/o Activity | 3 (2.2%) |
| Dimerization - Dimerization | 3 (2.2%) |
| Folding - Uncertain | 3 (2.2%) |
| Folding w/o Activity - Folding w/o Activity | 3 (2.2%) |
| Catalytic - Folding | 2 (1.5%) |
| Dimerization - Folding | 2 (1.5%) |
| Dimerization - Uncertain | 2 (1.5%) |
| Catalytic - Catalytic | 1 (0.7%) |
| Catalytic - No Protein | 1 (0.7%) |
| Folding - No Protein | 1 (0.7%) |
| Linker-2 - Uncertain | 1 (0.7%) |
| Included in Second Analysis: Participants with only one non-functioning allele (N=96) | |
| Catalytic - Dimerization | 28 (20.4%) |
| Catalytic - Folding | 24 (17.5%) |
| Catalytic - Uncertain | 10 (7.3%) |
| Catalytic - Catalytic | 7 (5.1%) |
| Dimerization - Folding w/o Activity | 6 (4.4%) |
| Folding w/o Activity - Uncertain | 6 (4.4%) |
| Catalytic - No Protein | 5 (3.6%) |
| Catalytic - Linker-2 | 3 (2.2%) |
| Folding - Folding w/o Activity | 3 (2.2%) |
| Folding - Linker-2 | 2 (1.5%) |
| Catalytic - Folding w/o Activity | 1 (0.7%) |
| Folding w/o Activity - No Protein | 1 (0.7%) |
| † The non-functioning alleles identified in the natural history are p.Arg141His, p.Phe157Ser, p.Asp188Gly, p.Thr237Met, or p.Thr237Arg. | |

**Supp. Table S5. Summary of Pathogenic Variants (N=137)**

| Variant | | | |  | | |
| --- | --- | --- | --- | --- | --- | --- |
| Short Name | HGVS Protein (NP_000294.1) | HGVS Nucleotide (NM_000303.3) | Category | All (N=137) | First Analysis Population† (N=88) | Second Analysis Population‡ (N=96) |
| R141H | p.Arg141His | c.422G>A | Catalytic | 80 (58.4%) | 67 (76.1%) | 77 (80.2%) |
| P113L | p.Pro113Leu | c.338C>T | Dimerization | 29 (21.2%) | 24 (27.3%) | 19 (19.8%) |
| F119L | p.Phe119Leu | c.357C>A | Dimerization | 17 (12.4%) | 12 (13.6%) | 12 (12.5%) |
| V231M | p.Val231Met | c.691G>A | Folding | 11 (8.0%) | 7 (8.0%) | 10 (10.4%) |
| C241S | p.Cys241Ser | c.722G>C | Uncertain | 11 (8.0%) | - | 8 (8.3%) |
| F157S | p.Phe157Ser | c.470T>C | Folding w/o Activity | 9 (6.6%) | 1 (1.1%) | 6 (6.3%) |
| R162W | p.Arg162Trp | c.484C>T | Uncertain | 9 (6.6%) | - | 3 (3.1%) |
| T237M | p.Thr237Met | c.710C>T | Folding w/o Activity | 9 (6.6%) | 4 (4.5%) | 3 (3.1%) |
| T237R | p.Thr237Arg | c.710C>G | Folding w/o Activity | 8 (5.8%) | 5 (5.7%) | 8 (8.3%) |
| V129M | p.Val129Met | c.385G>A | Folding | 7 (5.1%) | 6 (6.8%) | 6 (6.3%) |
| R123Q | p.Arg123Gln | c.368G>A | Catalytic | 5 (3.6%) | 1 (1.1%) | - |
| C9fs | p.Cys9AlafsTer27 | c.24delC | No Protein | 4 (2.9%) | 3 (3.4%) | - |
| E139K | p.Glu139Lys | c.415G>A | No Protein | 4 (2.9%) | 3 (3.4%) | 3 (3.1%) |
| D148N | p.Asp148Asn | c.442G>A | Folding | 4 (2.9%) | 2 (2.3%) | 1 (1.0%) |
| G214S | p.Gly214Ser | c.640G>A | Folding | 4 (2.9%) | 4 (4.5%) | 2 (2.1%) |
| N216I | p.Asn216Ile | c.647A>T | Catalytic | 4 (2.9%) | 4 (4.5%) | 4 (4.2%) |
| G15A | p.Gly15Ala | c.44G>C | Catalytic | 3 (2.2%) | 3 (3.4%) | 1 (1.0%) |
| A108V | p.Ala108Val | c.323C>T | Dimerization | 3 (2.2%) | 3 (3.4%) | 3 (3.1%) |
| I132T | p.Ile132Thr | c.395T>C | Folding | 3 (2.2%) | 3 (3.4%) | 3 (3.1%) |
| R21G | p.Arg21Gly | c.61C>G | Catalytic | 2 (1.5%) | 1 (1.1%) | - |
| V44A | p.Val44Ala | c.131T>C | Folding | 2 (1.5%) | 2 (2.3%) | - |
| Note: There are 60 unique variants identified in the study. † First Analysis Population includes all participants whose variants are in an activity category with at least 4 participants, with no variants in the uncertain category. ‡ Participants with only one of the non-functioning variants: p.Arg141His, p.Phe157Ser, p.Asp188Gly, p.Thr237Met, or p.Thr237Arg. § Variant not previously identified for the PMM2 gene. | | | | | | |

**Supp. Table S6. Summary of Pathogenic Variants by Country**

| Variant | Belgium (N=13) | Czech Republic (N=17) | France (N=19) | Italy (N=8) | Poland (N=9) | Portugal (N=7) | Spain (N=8) | The Netherlands (N=10) | US (N=46) |
| --- | --- | --- | --- | --- | --- | --- | --- | --- | --- |
| R141H | 9 (69.2%) | 12 (70.6%) | 8 (42.1%) | 8 (100.0%) | 7 (77.8%) | 2 (28.6%) | - | 5 (50.0%) | 29 (63.0%) |
| P113L | 3 (23.1%) | 12 (70.6%) | 3 (15.8%) | - | 1 (11.1%) | - | 3 (37.5%) | 1 (10.0%) | 6 (13.0%) |
| F119L | 2 (15.4%) | - | 1 (5.3%) | - | - | - | - | 5 (50.0%) | 9 (19.6%) |
| V231M | 2 (15.4%) | 1 (5.9%) | - | - | 5 (55.6%) | - | - | - | 3 (6.5%) |
| C241S | 2 (15.4%) | - | 3 (15.8%) | - | - | 1 (14.3%) | 2 (25.0%) | - | 3 (6.5%) |
| F157S | - | - | 4 (21.1%) | - | - | 2 (28.6%) | 1 (12.5%) | - | 2 (4.3%) |
| R162W | 1 (7.7%) | 1 (5.9%) | - | - | - | 4 (57.1%) | 1 (12.5%) | 2 (20.0%) | - |
| T237M | - | - | 5 (26.3%) | - | - | 2 (28.6%) | - | - | 2 (4.3%) |
| T237R | 2 (15.4%) | 1 (5.9%) | 1 (5.3%) | - | 1 (11.1%) | - | - | - | 3 (6.5%) |
| V129M | - | - | 2 (10.5%) | 2 (25.0%) | 1 (11.1%) | - | - | - | 2 (4.3%) |
| R123Q | - | - | - | - | - | - | 2 (25.0%) | 2 (20.0%) | 1 (2.2%) |
| C9fs | - | 3 (17.6%) | 1 (5.3%) | - | - | - | - | - | - |
| E139K | - | - | 2 (10.5%) | - | - | - | - | - | 2 (4.3%) |
| D148N | - | - | 1 (5.3%) | - | - | - | - | - | 3 (6.5%) |
| G214S | - | - | 2 (10.5%) | - | - | - | 2 (25.0%) | - | - |
| N216I | - | - | - | 3 (37.5%) | - | - | - | - | 1 (2.2%) |
| G15A | - | - | - | - | - | - | - | - | 3 (6.5%) |
| A108V | - | - | - | - | - | - | - | - | 3 (6.5%) |
| I132T | 1 (7.7%) | 1 (5.9%) | 1 (5.3%) | - | - | - | - | - | - |
| R21G | - | - | 1 (5.3%) | - | - | - | - | 1 (10.0%) | - |
| V44A | - | - | - | - | - | - | 2 (25.0%) | - | - |
| Note: There are 60 unique variants identified in the study. † Variant not previously identified for the PMM2 gene. | | | | | | | | | |

**Supp. Table S7. Summary of Pathogenic Variants by Region**

| Variant | | | |  | | |
| --- | --- | --- | --- | --- | --- | --- |
| Short Name | HGVS Protein (NP_000294.1) | HGVS Nucleotide (NM_000303.3) | Category | All (N=137) | EU (N=91) | US (N=46) |
| R141H | p.Arg141His | c.422G>A | Catalytic | 80 (58.4%) | 51 (56.0%) | 29 (63.0%) |
| P113L | p.Pro113Leu | c.338C>T | Dimerization | 29 (21.2%) | 23 (25.3%) | 6 (13.0%) |
| F119L | p.Phe119Leu | c.357C>A | Dimerization | 17 (12.4%) | 8 (8.8%) | 9 (19.6%) |
| V231M | p.Val231Met | c.691G>A | Folding | 11 (8.0%) | 8 (8.8%) | 3 (6.5%) |
| C241S | p.Cys241Ser | c.722G>C | Uncertain | 11 (8.0%) | 8 (8.8%) | 3 (6.5%) |
| F157S | p.Phe157Ser | c.470T>C | Folding w/o Activity | 9 (6.6%) | 7 (7.7%) | 2 (4.3%) |
| R162W | p.Arg162Trp | c.484C>T | Uncertain | 9 (6.6%) | 9 (9.9%) | - |
| T237M | p.Thr237Met | c.710C>T | Folding w/o Activity | 9 (6.6%) | 7 (7.7%) | 2 (4.3%) |
| T237R | p.Thr237Arg | c.710C>G | Folding w/o Activity | 8 (5.8%) | 5 (5.5%) | 3 (6.5%) |
| V129M | p.Val129Met | c.385G>A | Folding | 7 (5.1%) | 5 (5.5%) | 2 (4.3%) |
| R123Q | p.Arg123Gln | c.368G>A | Catalytic | 5 (3.6%) | 4 (4.4%) | 1 (2.2%) |
| C9fs | p.Cys9AlafsTer27 | c.24delC | No Protein | 4 (2.9%) | 4 (4.4%) | - |
| E139K | p.Glu139Lys | c.415G>A | No Protein | 4 (2.9%) | 2 (2.2%) | 2 (4.3%) |
| D148N | p.Asp148Asn | c.442G>A | Folding | 4 (2.9%) | 1 (1.1%) | 3 (6.5%) |
| G214S | p.Gly214Ser | c.640G>A | Folding | 4 (2.9%) | 4 (4.4%) | - |
| N216I | p.Asn216Ile | c.647A>T | Catalytic | 4 (2.9%) | 3 (3.3%) | 1 (2.2%) |
| G15A | p.Gly15Ala | c.44G>C | Catalytic | 3 (2.2%) | - | 3 (6.5%) |
| A108V | p.Ala108Val | c.323C>T | Dimerization | 3 (2.2%) | - | 3 (6.5%) |
| I132T | p.Ile132Thr | c.395T>C | Folding | 3 (2.2%) | 3 (3.3%) | - |
| R21G | p.Arg21Gly | c.61C>G | Catalytic | 2 (1.5%) | 2 (2.2%) | - |
| V44A | p.Val44Ala | c.131T>C | Folding | 2 (1.5%) | 2 (2.2%) | - |
| Note: There are 60 unique variants identified in the study. † Variant not previously identified for the PMM2 gene. | | | | | | |

**Supp. Table S8. Phenotypes of Six Subjects with Novel Variants**

| Subject ID | Subject 06020 | Subject 06024 | Subject 12007 | Subject 13009 | Subject 13014 | Subject 17009 |
| --- | --- | --- | --- | --- | --- | --- |
| **Variants** | | | | | | |
| Novel Variant | p.Arg238His | p.Phe68Cys | p.Ala233Argfs*100 | p.Pro20Leu | p.Tyr76His | p.Tyr64Ser |
| Other Variant | p.Arg141His | p.Arg141His | p.Pro113Leu | p.Pro113Leu | p.Thr237Met | p.Arg21Gly |
| **Demographics** | | | | | | |
| Age at Entry in Study | 8 | 1 | 8 | 14 | 12 | 43 |
| Sex | Male | Male | Female | Female | Female | Female |
| **NPCRS Total Scores** | | | | | | |
| Section 1 Subscore | 4 | 4 | 4 | 4 | 3 | 5 |
| Section 2 Subscore | 5 | 2 | 5 | 5 | 0 | 5 |
| Section 3 Subscore | 9 | 10 | 11 | 20 | 7 |  |
| Total Score | 18 | 16 | 20 | 29 | 10 | 10 |
| **Coagulation** | | | | | | |
| Antithrombin Activity | 1.18 | 0.31^L^ | 0.34^L^ | 0.17^L^ | 0.32^L^ | 0.51^L^ |
| Protein C Activity (%) |  |  | 43^L^ | 29^L^ | 31^L^ | 68^L^ |
| Factor XI Activity (%) | 78 | 40^L^ | 40^L^ | 31^L^ | 33^L^ | 102 |
| Factor IX (%) |  |  | 62 | 67 | 73 | 87 |
| **Glycosylation** | | | | | | |
| A-oligo/Di-oligo Transferrin Ratio | 0.006 | 0.19^H^ |  |  |  |  |
| Mono-oligo/Di-oligo Transferrin Ratio | 0.04 | 0.61^H^ |  |  |  |  |
| **Metabolic** | | | | | | |
| Insulin-like Growth Factor 1 (ng/mL) |  |  | 214.8 | 223 |  | 208.0528 |
| Insulin-like Growth Factor Binding Protein 3 (µg/L) |  |  | 52000^H^ |  |  |  |
| **Liver Function** | | | | | | |
| Alkaline Phosphatase (IU/L) | 131^L^ |  | 319.8 | 270^H^ | 232 |  |
| Alanine Transaminase (IU/L) | 11 |  | 37.2^H^ | 64^H^ | 123^H^ | 18 |
| Aspartate Aminotransferase (IU/L) | 30 |  | 45^H^ | 48^H^ | 64^H^ |  |
| Ceruloplasmin (µmol/L) |  |  | 3.0586 | 1.5666 |  |  |
| ^L^ = Result is below lower limit of normal. H = Result is above upper limit of normal. Upper and lower limits of normal are from the local labs where results were obtained. | | | | | | |

**Supp. Table S9. Non-functional Variants: Second Analysis Population**

| 2nd Variant | |  | Non-functional Variants | | | | |
| --- | --- | --- | --- | --- | --- | --- | --- |
| Category | Variant |  | p.Arg141His n = 77 | p.Phe157Ser n = 6 | p.Thr237Arg n = 8 | p.Thr237Met n = 3 | p.Asp188Gly n = 2 |
| **Dimerization** | | | | | | | |
| Dimerization | p.Ala108Val |  | 1 | - | 2 | - | - |
|  | p.Phe119Leu |  | 12 | - | - | - | - |
|  | p.Pro113Leu |  | 15 | - | 3 | 1 | - |
| **Folding** | | | | | | | |
| Folding | p.Asp148Asn |  | - | - | - | 1 | - |
|  | p.Asp223Asn |  | 2 | - | - | - | - |
|  | p.Gly214Ser |  | 2 | - | - | - | - |
|  | p.Gly57Arg |  | 1 | - | - | - | - |
|  | p.Ile132Thr |  | 3 | - | - | - | - |
|  | p.Ile153Thr |  | 2 | - | - | - | - |
|  | p.Leu32Arg |  | 1 | - | - | - | - |
|  | p.Tyr229Ser |  | 1 | - | - | - | - |
|  | p.Val129Met |  | 5 | 1 | - | - | - |
|  | p.Val231Met |  | 7 | - | 1 | - | 2 |
| **Other** | | | | | | | |
| Catalytic | p.Asn216Ile |  | 4 | - | - | - | - |
|  | p.Gln177His |  | - | 1 | - | - | - |
|  | p.Gly15Ala |  | 1 | - | - | - | - |
|  | p.Gly208Ala |  | 1 | - | - | - | - |
|  | p.Thr18Ser |  | 1 | - | - | - | - |
| Linker-2 | p.Gly186Arg |  | 1 | - | - | - | - |
|  | p.Phe183Ser |  | 2 | - | - | - | - |
| No Protein | 640-23A>G |  | 2 | - | - | - | - |
|  | IVS2+3A>T |  | 1 | - | - | - | - |
|  | p.Glu139Lys |  | 2 | 1 | - | - | - |
| Uncertain | p.Arg162Trp |  | 3 | - | - | - | - |
|  | p.Arg238His |  | 1 | - | - | - | - |
|  | p.Cys241Ser |  | 3 | 3 | 2 | - | - |
|  | p.Met126Thr |  | 1 | - | - | - | - |
|  | p.Phe68Cys |  | 1 | - | - | - | - |
|  | p.Pro113Ser |  | 1 | - | - | - | - |
|  | p.Tyr76His |  | - | - | - | 1 | - |
|  | | | | | | | |

**Supp. Table S10. Phenotypes of Six Subjects with Two Non-Functioning Variants**

| Mutations (Category) | p.Arg141His/p.Thr237Met (C-FWO) | | |  | p.Phe157Ser/p.Thr237Met (FWO-FWO) | | |
| --- | --- | --- | --- | --- | --- | --- | --- |
|  | Subject 05012 | Subject 13015 | Subject 13019 |  | Subject 01001 | Subject 14001 | Subject 14007 |
| **Demographics** | | | | | | | |
| Age at Entry in Study | 27 | 7 | 21 |  | 10 | 11 | 5 |
| Sex | Female | Male | Female |  | Male | Male | Female |
| **NPCRS Total Scores** | | | | | | | |
| Section 1 Subscore | 4 | 4 | 4 |  |  |  |  |
| Section 2 Subscore | 5 | 1 | 3 |  |  |  |  |
| Section 3 Subscore | 10 | 9 | 14 |  |  |  |  |
| Total Score | 19 | 14 | 21 |  |  |  |  |
| **Coagulation** | | | | | | | |
| Antithrombin Activity | 0.5^L^ | 0.344^L^ | 0.29^L^ |  | 0.7^L^ |  |  |
| Protein C Activity (%) | 77 | 28^L^ | 25^L^ |  | 77 |  |  |
| Factor XI Activity (%) | 52^L^ | 25^L^ | 42^L^ |  |  |  |  |
| Factor IX (%) | 95 | 61 | 69 |  | 122.8 |  |  |
| **Glycosylation** | | | | | | | |
| A-oligo/Di-oligo Transferrin Ratio |  |  |  |  | 0.036^H^ |  |  |
| Mono-oligo/Di-oligo Transferrin Ratio |  |  |  |  | 0.37^H^ |  |  |
| **Metabolic** | | | | | | | |
| Insulin-like Growth Factor 1 (ng/mL) | 276 | 73.7^L^ | 238 |  | 155 | 140 |  |
| Insulin-like Growth Factor Binding Protein 3 (µg/L) | 63000 |  |  |  | 3900 | 41200 |  |
| **Liver Function** | | | | | | | |
| Alkaline Phosphatase (IU/L) | 59 | 273 | 86 |  | 252 | 109^L^ |  |
| Alanine Transaminase (IU/L) | 31 | 32 | 57^H^ |  | 68^H^ | 23 |  |
| Aspartate Aminotransferase (IU/L) | 27 | 42 | 32 |  | 46^H^ | 18 |  |
| Ceruloplasmin (µmol/L) | 2.6856 |  | 1.2682 |  |  | 2.1261 |  |
| ^L^ = Result is below lower limit of normal. H = Result is above upper limit of normal. Upper and lower limits of normal are from the local labs where results were obtained. | | | | | | | |
